# Regulatory adaptation of an accessory gene controls fungal halotolerance and niche expansion

**DOI:** 10.1093/ismejo/wrag162

**Published:** 2026-06-26

**Authors:** Mingyue Ding, Guan Pang, Mengting Huang, Yujie Shi, Fan Yi, Paolo Wong, Dongxin Shi, Zhilin Yuan, Lanxi Su, Junjie Guo, Weixiang Wang, Oded Yarden, Feng M Cai, Irina S Druzhinina, Qirong Shen

**Affiliations:** Jiangsu Provincial Key Lab of Solid Organic Waste Utilization, Key Lab of Organic-based Fertilizers of China, Jiangsu Collaborative Innovation Center of Solid Organic Wastes, Educational Ministry Engineering Center of Resource-saving Fertilizers, Nanjing Agricultural University, Nanjing 210095, China; College of Horticulture, Nanjing Agricultural University, Nanjing 210095, China; Jiangsu Provincial Key Lab of Solid Organic Waste Utilization, Key Lab of Organic-based Fertilizers of China, Jiangsu Collaborative Innovation Center of Solid Organic Wastes, Educational Ministry Engineering Center of Resource-saving Fertilizers, Nanjing Agricultural University, Nanjing 210095, China; Jiangsu Provincial Key Lab of Solid Organic Waste Utilization, Key Lab of Organic-based Fertilizers of China, Jiangsu Collaborative Innovation Center of Solid Organic Wastes, Educational Ministry Engineering Center of Resource-saving Fertilizers, Nanjing Agricultural University, Nanjing 210095, China; State Key Laboratory of Biocontrol, School of Ecology, Shenzhen Campus of Sun Yat-sen University, Sun Yat-sen University, Shenzhen 518107, China; State Key Laboratory of Biocontrol, School of Ecology, Shenzhen Campus of Sun Yat-sen University, Sun Yat-sen University, Shenzhen 518107, China; State Key Laboratory of Biocontrol, School of Ecology, Shenzhen Campus of Sun Yat-sen University, Sun Yat-sen University, Shenzhen 518107, China; Research Institute of Subtropical Forestry, Chinese Academy of Forestry, Hangzhou 311400, China; Spice and Beverage Research Institute, Chinese Academy of Tropical Agricultural Sciences, Wanning 571533, China; State Key Laboratory of Biocontrol, Guangdong Provincial Key Laboratory of Plant Stress Biology, School of Agriculture and Biotechnology, Shenzhen Campus of Sun Yat-sen University, Sun Yat-sen University, Shenzhen 518107, China; Beijing Key Laboratory of New Technology in Agricultural Application, Beijing University of Agriculture, Beijing 102200, China; Department of Plant Pathology and Microbiology, the Robert H. Smith Faculty of Agriculture, Food and Environment, The Hebrew University of Jerusalem, Rehovot 7612001, Israel; State Key Laboratory of Biocontrol, School of Ecology, Shenzhen Campus of Sun Yat-sen University, Sun Yat-sen University, Shenzhen 518107, China; Taxonomy and Systematics, Royal Botanic Gardens, Kew, Richmond, TW9 3DS, United Kingdom; Jiangsu Provincial Key Lab of Solid Organic Waste Utilization, Key Lab of Organic-based Fertilizers of China, Jiangsu Collaborative Innovation Center of Solid Organic Wastes, Educational Ministry Engineering Center of Resource-saving Fertilizers, Nanjing Agricultural University, Nanjing 210095, China

**Keywords:** coastal species, glycerol dehydrogenase, halotolerance, niche expansion, osmotic stress, synthetic community, *Trichoderma asperelloides*

## Abstract

Stress tolerance underpins ecological plasticity and niche expansion in fungi. Although *Trichoderma* is best known from sylvan, mycoparasitic, soil-, and plant-associated habitats, its occasional recovery from saline soils raises questions about the molecular basis of this adaptation. A coastal survey revealed limited but persistent *Trichoderma* diversity with frequent recovery of halotolerant *Trichoderma asperelloides*. Screening a salt-stressed *T. asperelloides* cDNA library in *Saccharomyces cerevisiae* identified 62 inserts, of which 12 were salt responsive *in vivo*. Among then, deletion of *gld1*, encoding an aldo-keto reductase, impaired halotolerance and glycerol accumulation. In a 27-species synthetic community, the Δ*gld1* mutant was competitively displaced by other species under salinity. Cross-species promoter replacement in a salinity-sensitive strain *Trichoderma atroviride* increased its halotolerance. A global *tef1* haplotype network placed the coastal isolates within broader *T. asperelloides* diversity, consistent with recurrent expansion into saline/coastal soils. Together, these findings link accessory-gene regulation to niche expansion in fungi.

## Graphical Abstract

**Graphical Abstract:**
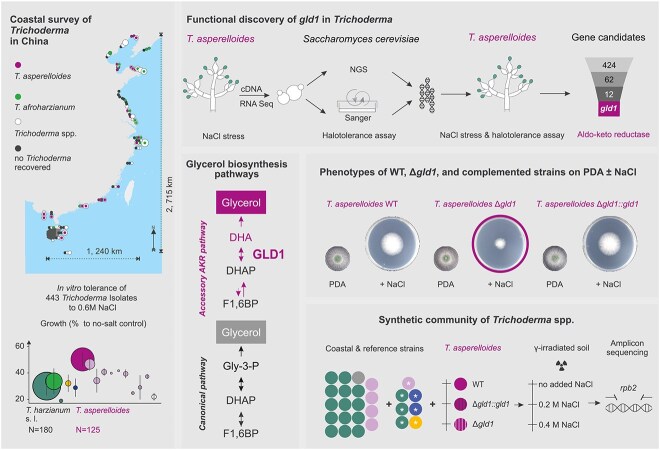

## Introduction

Fungi exhibit remarkable ecological plasticity, with many lineages expanding into disturbed or extreme environments [1, 2]. Salinity imposes some of the strongest physiological constraints in terrestrial habitats, leading to osmotic imbalance, ionic toxicity, and reactive oxygen species (ROS) accumulation [3, 4]. Yet some fungi persist in saline environments through genetic and physiological adaptations, making them key models for stress biology and resilience [5–7].

Although *Trichoderma* (*Hypocreales, Ascomycota*) is best known as a sylvan, mycoparasitic, and plant-associated genus, some species exhibit remarkable environmental opportunism [8, 9]. Many of them were recovered from diverse transitional and potentially extreme habitats, including rhizospheres, plant endospheres, burned forests, indoor and clinical settings, and even salt-affected soils and marine substrates [9–11]. This broad distribution likely reflects repeated niche expansions over evolutionary time [12]. About ten percent of *Trichoderma* species such as *Trichoderma harzianum, Trichoderma afroharzianum, Trichoderma atroviride, Trichoderma asperellum, Trichoderma virens*, and others exhibit polyextremotolerance and act as immunizing commensals of crops [8, 13], supporting their widespread use as biofertilizers and biocontrol agents, particularly in stress-prone soils [10, 14].

Despite widespread environmental sequencing and over 500 known species [11, 15], *Trichoderma* is rarely detected in natural soils beyond agriculturally disturbed habitats, and no cryptic reservoir of uncultured diversity has been detected in soil [11]. The species most frequently recovered—such as those listed above—are the same cohort routinely cultured, studied, and applied in agriculture [8, 9]. This convergence highlights a biologically relevant, ecologically opportunistic set of frequently unrelated species that dominates both agricultural and soil-isolated communities. As functional genomics requires *in vitro* cultivation, these species offer a unique opportunity to experimentally dissect fungal adaptive traits and their real-world environmental persistence.

The mechanistic basis of environmental opportunism in *Trichoderma* may involve several genomic features, including expansions of small secreted cysteine-rich proteins (SSCPs) [16], regulatory gene networks [9], and laterally acquired carbohydrate-active enzymes [17], but remains largely unresolved. Comparative genomics highlights these and other gene families—such as membrane transporters and signalling proteins—as potential contributors to adaptive plasticity [8, 9]. Yet it remains unclear whether the occurrence of some *Trichoderma* species in atypical environments reflects genuine niche expansion, transient colonization, or sampling artifacts. Disentangling these alternatives may provide a much needed understanding of how genome architecture underpins ecological breadth and fungal persistence.

Osmotolerance is a core physiological adaptation trait that enables fungi to persist and proliferate across a wide range of challenging environments [4, 18]. In *Trichoderma*, this includes not only saline soils, but also decaying or burned wood, the rhizosphere, mycoparasitic, endophytic, marine, and even human-associated habitats—each imposing osmotic or ionic stress due to fluctuating water activity, solute accumulation, or host-derived defenses [10]. Fungal osmoregulation typically relies on intracellular glycerol biosynthesis and retention, which stabilizes proteins and membranes under hyperosmotic conditions [4, 5]. Filamentous fungi use two main glycerol-producing routes: a primary pathway involving glycerol-3-phosphate dehydrogenase (GPD) and glycerol-3-phosphatase (GPP) [4], and a so-called “alternative” dihydroxyacetone (DHA) pathway mediated by NADP^+^-dependent aldo-keto reductases such as GLD1/2 [19–21]. Although conserved across fungal lineages, these pathways vary in regulation, capacity, and ecological deployment. In *Aspergillus nidulans (Eurothiales, Ascomycota)*, GLD proteins contribute to osmoadaptation [19], and in *T. atroviride* [20] and *Trichoderma reesei* [21], salt shock-responsive regulation has been reported despite limited halotolerance. Other fungi rely on diverse strategies, from glycerol hyperaccumulation to melanin-mediated ion exclusion and Na^+^ efflux [5–7, 22]. Yet, few studies link osmoregulatory gene function to environmental persistence or niche expansion under field-relevant conditions.

Here, we investigate the ecological distribution and genetic basis of halotolerance in *Trichoderma* along a 2700 km coastal transect spanning China’s four major sea regions. Although we recovered 443 *Trichoderma* isolates, saline soils yielded limited but persistent cultivable diversity, with frequent recovery of *Trichoderma asperelloides*, a cryptic species sister to the commonly seen species *T. asperellum* [23]. To identify halotolerance-related genes, we performed a heterologous screen of a salt-induced *T. asperelloides* cDNA library in *Saccharomyces cerevisiae*, coupled with high-throughput sequencing and Sanger sequencing validation. Of 62 yeast-identified genes, 12 were transcriptionally induced under salt stress in *T. asperelloides* and selected for gene deletion. Deletion of *gld1*, encoding an aldo-keto reductase involved in accessory glycerol biosynthesis, impaired halotolerance and glycerol accumulation. In a synthetic *Trichoderma* community established in γ-irradiated forest soil amended with NaCl, the Δ*gld1* mutant was displaced under salinity, as shown by high-resolution *rpb2*-based DNA metabarcoding. Together, these results identify *gld1* as a functional determinant of osmotic fitness in *T. asperelloides* and illustrate how accessory stress-response pathways can support fungal niche expansion under saline conditions.

## Materials and methods

### Environmental sampling and fungal cultivation

#### Sample collection and chemical soil profiling

Coastal saline soil samples (*n* = 83) were collected from 29 locations spanning China's four major sea regions (Bohai Sea, Yellow Sea, East China Sea, and South China Sea; E109°7′-122°41′, N16°49′-40°53′). Surface soils (0–20 cm depth) were collected using stainless steel corers, with GPS coordinates recorded. Samples were transported to the laboratory on ice, then homogenized, and divided into two aliquots: one for fungal screening (stored at 4°C), and the other for chemical analysis (air dried). For soil pH and electrical conductivity (EC), each air-dried sample was first passed through a 20-mesh sieve and then mixed with deionized water (1:5, w/v). The supernatants were then measured using a multifunction pH meter (Sartorius PB-10, Germany). Contents of total carbon and total nitrogen of each sample (*n* = 3) were determined using an elemental analyzer (Vario EL III, Germany) with oven-dried samples that were ground to <100 μm.

#### *Trichoderma* isolation, identification and salt tolerance determination

Each soil sample was suspended in sterile water (1 g in 9 ml) and subjected to the spread plate method on 9-cm potato dextrose agar (PDA) plates to selectively recover *Trichoderma* [14]. Pure cultures were molecularly identified following the authoritative guidelines for *Trichoderma* molecular identification [15]. Internal Transcribed Spacers 1 and 2 of the rRNA gene cluster (ITS) was used to confirm placement in *Trichoderma* genus, whereas species-level assignments were based on the concordance of partial genetic markers ITS, translation elongation factor 1 encoding gene *tef1,* and RNA polymerase subunit B II encoding gene *rpb2*. To assess salt tolerance, all recovered isolates and reference strains (Table 1) were cultured on PDA supplemented with 0.6 or 1.2 M NaCl, alongside unsupplemented PDA as control. Relative growth rate (RGR) under salt stress was calculated as:


$$\mathrm{RGR}\ \left(\%\right)=\left(\mathrm{Ds}/\mathrm{Dc}\right)\times 100$$

where Ds and Dc are colony diameters under salt-added and no-added conditions, respectively. The list of all isolates used in this study, NCBI GenBank accession numbers, and salt tolerance are provided in Supplementary Data 1.

**Table 1:** *Trichoderma* strains used in this study and their experimental deployment

*Trichoderma* taxon		Strain ID	Strain category	Halotolerance Assay	Microcosm	n
Total				508	29	508
*Viride* clade	*T. asperelloides*	X01119	Parental coastal isolate	+	X01119	1
		X01466–X01467	Deletion: *Δgld1*	+	*Δgld1*	2
		X01468	Complemented: *Δgld1::gld1*	+	*Δgld1::gld1*	1
		X01469–X01470	Deletion: *Δakr2a*	+	−	2
		X01471–X01472	Deletion: *Δakr2b*	+	−	2
		X01473–X01474	Deletion: *Δgpd1*	+	−	2
		X01475–X01476	Deletion: *Δgpp1*	+	−	2
		X01477	Deletion: *Δhog1*	+	−	1
		X01444–X01445	Deletion: *ΔKAL9468950*	+	−	2
		X01446–X01447	Deletion: *ΔKAL9469123*	+	−	2
		X01448–X01449	Deletion: *ΔKAL9484779*	+	−	2
		X01450–X01451	Deletion: *ΔKAL9476955*	+	−	2
		X01452–X01453	Deletion: *ΔKAL9477209*	+	−	2
		X01454–X01455	Deletion: *ΔKAL9485363*	+	−	2
		X01456–X01457	Deletion: *ΔKAL9480109*	+	−	2
		X01458	Deletion: *ΔKAL9483789*	+	−	1
		X01459–X01460	Deletion: *ΔKAL9469028*	+	−	2
		X01461–X01462	Deletion: *ΔKAL9471413*	+	−	2
		X01463–X01464	Deletion: *ΔKAL9473491*	+	−	2
		X01465	Deletion: *ΔKAL9479765*	+	−	1
		X01067–X01191	Coastal isolates	+	−	124
		X01479–X01488	Non-coastal isolates	+	−	10
		**Tr.10**	Reference strain	+	−	1
		**SZMC 27998**	Reference strain	+	−	1
	*T. asperellum*	**CBS 433.97**	Reference strain	+	−	1
		X01192–X01209	Coastal isolates	+	X01193	18
		X01489–X01494	Non-coastal isolates	+	−	6
	*T.* cf. *asperellum* PS1	X01315–X01320	Coastal isolates	+	−	6
	*T. arenarium*	**TUCIM 10301**	Reference strain	+	−	1
		X01065–X01066	Coastal isolates	+	X01065	2
	*T. atroviride*	**IMI 206040**	Reference strain	+	IMI 206040	1
		X01478	Promoter swap: P*_Tasp_gld1::gld1*	+	−	1
		X01210–X01216	Coastal isolates	+	−	7
	*T*. cf. *atroviride* PS1	X01321–X01322		+	−	2
	*T.* cf. *obovatum*	X01240		+	−	1
	*T.* cf. *uncinatum*	X01256		+	−	1
	*T.* cf. *vadicola*	X01257–X01259		+	−	3
	*T. hongkuii*	X01284–X01288		+	X01286	5
	*T. nigricans*	X01289–X01294		+	−	6
	*T. parahamatum*	X01295–X01297		+	X01295	3
		X01495	Non-coastal isolates	+	−	1
	*T. hamatum*	X01496	Non-coastal isolates	+	−	1
*T. harzianum* species complex	*T. harzianum*	**CBS 226.95**	Reference strain	+	CBS 226.95	1
		X01277–X01283	Coastal isolates	+	−	7
	*T.* sp. NJAU 4742	**NJAU 4742**	Reference strain	+	NJAU 4742	1
	*T.* cf. *breve*	X01222–X01237	Coastal isolates	+	X01234	16
	*T.* cf. *lentinulae*	X01238–X01239		+	X01238	2
	*T.* cf. *pholiotae*	X01241–X01255		+	X01245	15
	*T.* cf. *zelobreve*	X01260–X01276		+	−	17
	*T. shaanxiensis*	X01298–X01314		+	X01306	17
	*T.* cf. *bannaense* PS1	X01323		+	X01323	1
	*T.* cf. *guizhouense* PS1	X01326–X01331		+	−	6
	*T.* cf. *guizhouense* PS2	X01332–X01341		+	X01332	10
	*T.* cf. *pingquanense* PS2	X01345–X01364		+	X01355	20
	*T.* cf. *pyramidale* PS3	X01365–X01390		+	X01380	26
	*T.* cf. *guizhouense* PS3	X01391–X01394		+	X01392	4
	*T.* cf. *guizhouense* PS4	X01395–X01402		+	X01397	8
	*T.* cf. *guizhouense* PS5	X01403–X01411		+	X01409	9
	*T.* cf. *guizhouense* PS6	X01436–X01437		+	−	2
	*T.* cf. *simile* PS1	X01412–X01415		+	X01415	4
	*T.* cf. *simile* PS2	X01416–X01421		+	−	6
	*T.* cf. *simile* PS3	X01422–X01426		+	−	5
	*T.* cf. *simile* PS4	X01427		+	−	1
	*T.* cf. *simmonsii* PS1	X01428–X01431		+	X01430	4
*Harzianum* clade	*T. afroharzianum*	**T–22**	Reference strain	+	T-22	1
		X01000–X01064	Coastal isolates	+	−	65
	*T.* cf. *miyunense* PS1	X01342–X01344		+	X01343	3
*Virens* clade	*T. virens*	**Gv 29-8**	Reference strain	+	Gv 29-8	1
		X01438–X01442	Coastal isolates	+	−	5
	*T*. cf. *catoptron* PS1	X01324–X01325		+	−	2
Section *Longibrachiatum*	*T. reesei*	**CBS 999.97**	Reference strain	+	−	1
		**QM 6a**		+	QM 6a	1
	*T. longibrachiatum*	**ATCC18648**		+	ATCC 18648	1
	*T.* cf. *xanthum* PS1	X01432–X01435	Coastal isolates	+	−	4
*Brevicompactum* clade	*T. brevicompactum*	X01217–X01221	Coastal isolates	+	X01218	5

Strain identifiers, taxonomic affiliations, and experimental roles are summarized for all 508 *Trichoderma* strains used in halotolerance assays (*n* = 508) and for the 27 species/29 strains included in the synthetic microcosm experiment. Coastal isolates from this study, gene deletion and promoter swap mutants, complemented strains, and widely used reference strains (in bold) are included. Strains are categorized by clade, with representative phylotypes spanning the *Viride, Harzianum, Longibrachiatum, Brevicompactum*, and *Virens* infrageneric groups. For each group, deployment in halotolerance assays and/or the microcosm is indicated. Mutant strains include deletions of *gld1, akr2a, akr2b, gpd1, gpp1, hog1*, and salt-induced candidate genes identified via cDNA library screening (Fig. 2). PS, putative phylogenetic species.

### Molecular biology techniques

#### *Trichoderma* cDNA library construction and high-throughput screening for salt-responsive genes

To mine out the key halotolerance gene(s), a *Trichoderma* cDNA library was constructed using pYES2 plasmid (Invitrogen, ThermoFisher Scientific, USA). To do so, a representative strain (*T. asperelloides* X01119) was grown in a shaking culture (170 rpm and 25°C) in potato dextrose broth (PDB, Difco, USA) supplemented with 0.8 M NaCl, for 72 h. The total RNA was extracted from the biomass using the SteadyPure Plant RNA Kit (Accura, China). The mRNA was purified using oligo(dT) magnetic beads then used for three-frame cDNA library synthesis. The resulting cDNA underwent amplification, purification, normalization, and small-fragment removal. Double-stranded cDNA was then ligated into the pYES2 expression vector that was constructed with the yeast inducible *GAL1* promoter (by galactose) and the URA3 selection marker (Invitrogen). The recombinant plasmids were transformed into *Escherichia coli* TOP10 competent cells (Vazyme, China). The plasmids were amplified by liquid culture of all of the growing *E. coli*. Then the purified library plasmid DNA was transformed into the recipient strain *S. cerevisiae* INVSc1 with the LiAc-mediated yeast transformation procedure following the Clontech manual (PT3024-1, Takara, Japan). A transformant harboring the empty pYES2 vector was generated in parallel to serve as the control for subsequent gene screening assays. The obtained yeast clone library was screened at 1.3 M NaCl-added SG-U medium (see recipes in Yeast Protocols Handbook, PT3024-1, Takara, Japan). Illumina sequencing (Hiseq 2500) was then used for determining the genes with function of enhanced salt tolerance in yeast. Genes detected were further screened by replating the corresponding clone strain on SG-U medium with NaCl added and also for the expression pattern of potential genes in the natural host (*T. asperelloides* X01119) by quantitative polymerase chain reaction (qPCR).

#### Gene expression profiling

Reverse-transcriptional qPCR was used to determine the relative expression of genes of interest. For each cDNA synthesis reaction, 1 μg total RNA extracted by using the SteadyPure Plant RNA Kit (Accura, China) was reverse-transcribed using HiScript II Q RT SuperMix (Vazyme, China), according to the manufacturer’s instructions. qPCR was carried out in a thermal cycler qTOWER3 (Jena Analytics, Germany). Briefly, each 20 μl reaction mixture contained 100 ng of cDNA, 0.5 μM of each primer, and 10 μl of ChamQ Universal SYBR qPCR Master Mix (Vazyme, China). The thermal cycle process included an initial cycle at 95°C for 30 s, followed by 40 cycles at 95°C for 3 s and 60°C for 15 s. The *tef1* was used as the housekeeping gene for qPCR. The relative transcript abundance of each gene is calculated according to the 2^−∆CT^ method as reported previously [16]. All primers are listed in Supplementary Data 2.

#### Targeted gene deletion, gene complementation, and promoter swap

Targeted gene deletions were carried out in the wild-type strain (WT) of *T. asperelloides* X01119, as reported previously [24]. Briefly, to construct the gene deletion vector, ca. 2 kb of the 5′- and 3′- flanking arms of the open reading frame (ORF) of the target gene were amplified and fused to a hygromycin B resistance cassette (*hph*). The construction was then used for gene replacement via polyethylene glycol (PEG)-mediated protoplast transformation [25]. For gene complementation of the deletion strain, a WT gene copy (ca. 1 kb upstream and 1 kb downstream of the ORF) was fused with a fluorescent tag cassette (eGFP at the C-terminus of the gene) by PCR and transformed into the protoplasts of the deletion mutant lacking the corresponding gene. To replace the promoter of *gld1* gene in a halosensitive strain *T. atroviride* IMI 206040, a copy of ca. 1.5 kb upstream of the *gld1* ORF from T. asperelloides X01119 was inserted, with a *hph* cassette fused at the 5′-end of the promoter region. Two 2-kb flanking arms upstream and downstream of the designated promoter region were also fused to the promoter swap vector to prime homologous recombination. Positive transformants were first confirmed by PCR, and further validated by Sanger sequencing. Mutants were maintained on PDA plates containing hygromycin B (300 μg ml^−1^, Solarbio, China) contained.

### Phenotyping and biochemical assays

#### Growth under five stress conditions

To test whether the targeted gene/pathway is involved in response to other stress factors, five stress conditions including two ionic/osmotic stressors (NaCl and KCl), two non-ionic osmotic stressors (glycerol and sorbitol), and one oxidative stress (H_2_O_2_), each applied across five concentration gradients, using those grown on PDA as the control. The strains were cultivated on plates for 60 h at 25°C in darkness. The colony growth under each condition per each strain was recorded by a Canon EOS 70D (equipped with a Canon 100 mm macro lens) under white light. qPCR was used to determine the transcriptional level of genes involved in glycerol metabolism, as detailed above.

#### Enzymatic and metabolic assays

In order to monitor glycerol metabolism in response to the presence of salt, a time course experiment determining the target enzymatic activity in *Trichoderma* mycelia was carried out. Spores of each strain (1 × 10^7^ cells ml^−1^) were first cultured in shaking PDB at 25°C and 170 rpm, for 48 h. Mycelia were then transferred to PDB amended with 0.8 M NaCl and sampled at 0, 4, 6, 8, and 24 h after exposure to salt. The crude enzyme extracts were prepared by homogenizing the mycelia in a buffer containing 50 mM KH₂PO₄, 5 mM MgCl_2_ and 0.5 mM EDTA (pH = 7.0) at 4°C. Then, the oxidase/reductase activities of the extracts were determined by monitoring NADPH at 340 nm in a microplate reader as reported [19, 21]. Dehydrogenase activity was measured in 200 mM Tris–HCl (pH = 9.5) containing 1 mM NADP^+^ (Beyotime, China) and 10 mM glycerol (Sigma, USA) and reductase activity was determined in 10 mM Na_3_PO_4_ (pH = 7.0) containing 400 μM NADPH (Beyotime, China) and 10 mM dihydroxyacetone (DHA, Sigma, USA). The protein concentration of each crude enzyme extract was measured with the BCA Protein Quantification Kit (Vazyme, China) according to the manufacturer’s instructions. The intracellular glycerol content per each strain was quantified using the Glycerol Assay Kit (Enzyme Method, G0912W, Grace Biotechnology Co., Ltd, China) with a time course from 0–96 h after salt exposure.

To measure Na^+^ flux in each genotype when exposed to salt stress, the non-invasive micro-test technology (NMT, YoungerUSA, LLC, Amherst, USA) was adopted to detect the net ion flux into or out of living hyphae *in situ*. To do so, 48-h-old hyphae grown on PDA plates were collected and treated with the measuring buffer (0.8 M NaCl, 1 mM KCl, and 0.2 mM MES, pH 5.6) for 1, 3, and 48 h. Following the calibration of the microsensor, ion fluxes were recorded for 5 min in the zone 5 μm apart from the hyphal clump.

### Omics study and bioinformatics

#### Whole-genome sequencing and assembly

Genomic DNA (gDNA) of *T. asperelloides* X01119 was extracted using a plant DNA extraction kit from Qiagen (DNeasy Plant Mini Kit, USA), following the manufacturer’s protocol. The obtained gDNA was then sequenced using a combination of PromethION (Oxford Nanopore Technologies, Oxford, UK) and NovaSeq 6000 System (Illumina, USA) platforms. The whole-genome sequence of X01119 was *de novo* assembled by using NECAT, followed by iterative polishing using Racon 1.4.13 [26] and Pilon 1.23 [27] to fill gaps and correct local mis-assemblies. Genome assembly consistency was assessed by aligning Illumina reads to the assembled genome using BWA (v0.7.17) [28], with mapping rate and coverage depth calculated for evaluation. Assembly completeness was evaluated using BUSCO (v4.1.2) [29]. Gene prediction integrated three approaches including homology-based prediction by Exonerate (v2.4.0), de novo prediction by Augustus (v3.3.2) and GlimmerHMM (v3.0.4), and transcript-based prediction using TransDecoder (v5.1.0), followed by the final integration with MAKER (v2.31.10). Protein sequences were functionally annotated through sequence similarity by DIAMOND BLASTp (v2.0.11.149; e-value ≤1.0E-5) against NR, UniProt, and KEGG databases [30]. KOBAS (v3.0) mapped KEGG Orthology (KO) identifiers to metabolic pathways. Protein family/motif annotation was given by InterProScan (v5.5.2-86) [31]. Protein family classification was generated via HMMER hmmscan (v3.3.2; e-value ≤0.01) [32] against the Pfam database.

#### Transcriptomic assay

To prepare the mRNA library response to osmotic shock (PDB + 0.8 M NaCl), *T. asperelloides* X01119 was first cultured in shaking PDB as mentioned above for 48 h, then transferred to PDB amended with 0.8 M NaCl. Fungal biomass was sampled at 0, 5, 15, 30, 60 and 120 min after salt exposure. The harvested mycelia were immediately frozen in liquid nitrogen, and RNA extracted and sent for Illumina sequencing of corresponding cDNA (HiSeq System, PE150) in Wuhan Benagen Tech. Co. Ltd., China. The obtained clean reads were mapped against the genome of *T. asperelloides* X01119 using HISAT2 (v2.2.1) [33] and the reads amount mapped to each gene was counted using HTSeq (v0.13.5) [34]. The differentially expressed gene (DEG) analyses based on the matrix of counts were performed using the R package DESeq2 [35]. Genes characterized by |log2foldchange| > 1, and adjusted *P*-value <.05 were considered as the DEGs.

#### Phylogenetic analysis and natural selection pressure calculation

For AKR gene family phylogeny, the amino acid sequences of target proteins were retrieved from a local database comprising 142 representative fungal genomes selected from public databases of NCBI and the JGI Genome portal (https://genome.jgi.doe.gov/portal/) via BLASTp (e-value ≤1.0E-5, identity cutoff ≥35%) [36], using the ones from *T. asperelloides* X01119 genome as the query sequence. Multiple sequence alignment was performed using MUSCLE 3.8.31 [37]. Maximum likelihood (ML) phylogenetic tree was generated using IQ-TREE 2.3.0 [38] with the best-fit substitution model selected based on the Bayesian Information Criterion by ModelFinder [39]. Statistical support was inferred by 1000 bootstrapping replicates. The three-locus ML tree for *Trichoderma* strains studied in the current work was reconstructed by using ITS, *tef1* and *rpb2*, as recommended for this genus [15].

To test for possible adaptive evolution of *gld1* in halotolerant lineages, codon alignments were analyzed using CodeML in PAML 4 [40]. The dataset included *gld1* sequences from seven representative *Trichoderma* species: *Trichoderma longibrachiatum, T. harzianum, T. afroharzianum, T. virens, T. asperelloides, T. asperellum*, and *T. atroviride*. Branch-site model A was used (model = 2, NSsites = 2) and compared with the corresponding null model in which the foreground positive-selection class was fixed at neutral evolution (fix_omega = 1, omega = 1). The alternative model allowed four codon classes: conserved sites with ω₀ estimated from the data, neutral sites with ω₁ fixed at 1, and two foreground classes in which ω₂ was estimated and allowed to exceed 1. Foreground branches were tested in separate runs for all seven lineages. Likelihood ratio tests (LRTs) were used to compare the alternative and null models. Tree files and alignments are provided in Supplementary Data 3.

To compare regulatory architecture of *gld1*, 1.5-kb upstream regions were extracted from *T. asperelloides* X01119 and representative *T. asperelloides* strains for which promoter sequences were available, together with the corresponding *T. atroviride* promoter. Sequences were oriented relative to the *gld1* start codon and manually checked. Ungapped motifs enriched in the *T. asperelloides* promoter set were identified using STREME [41]. Motif presence and position were then mapped across the aligned promoter sequences. Motif sequences, coordinates, and presence/absence scoring are provided in Supplementary Data 4.

#### *rpb2* gene amplicon sequencing of synthetic *Trichoderma* community in microcosm

In order to see the impact of target gene deletion on the competitive colonization of saline soil, a synthetic community microcosm was carried out with 29 strains (=27 representative species) from the *Trichoderma* genus. Specifically, conidia suspension containing equally represented spores (10^7^ spores ml^−1^) from each strain was inoculated into a γ-radiation sterilized soil with NaCl added. To avoid the effect from other potential constraining conditions on *Trichoderma* growth, we adopted an organic-enriched forest soil (Entisols, from Nanjing, China) to do this experiment. The soil was amended with NaCl at a concentration of either 0, 0.2, or 0.4 M. After 10 days of incubation at 25°C and at 70% relative humidity, the fungal community composition was assessed via a customized *rpb2* amplicon sequencing method by NovaSeq 6000 System (Illumina) that targets a fragment of the *rpb2* (~380 bp) specifically designed for DNA barcoding distinguishing *Trichoderma* spp. at the species level. The primer pair used were ComRPB2-F and ComRPB2-R (Supplementary Data 2). Amplicon sequence data were processed and analyzed by QIIME 2 [42]. The “cutadapt” and “dada2” plugins were respectively used for removing the adaptors and primers of sequences, and performing the quality control and assignment of amplicon sequence variants. The taxonomic assignment was given by the “BLAST” plugin against the local *Trichoderma rpb2* database that includes reference sequences from the 27 species used, with the cutoff of identity ≥0.99 and coverage ≥0.95. The taxonomic assignment robustness was tested by introducing 1–24 substitutions into randomly selected reference sequences at polymorphic sites, with 1000 simulations per mutation level, followed by reassignment against a 146-species *rpb2* local reference database and 0.99 cutoffs for the amplicon data. The reference alignment, pairwise identity matrix, simulation outputs, and reassignment results are provided in Supplementary Data 5. To independently validate the amplicon-derived community profiles, species/strain-specific TaqMan probe qPCR assays targeting partial *rpb2* were developed for eight representative species in the microcosm.

#### Absolute qPCR of fungal DNA in microcosm

In order to compare the total fungal biomass in each microcosm soil across samples, the total gDNA from each sample was further examined for absolute qPCR analysis that quantifies the *rpb2* copy numbers per each gram of substrate. The qPCR reaction mixture and the thermal cycling program were conducted as detailed above. The standard curve for absolute qPCR was generated by using the *rpb2* fragment (see primers in Supplementary Data 2) from the strain *Trichoderma* sp. NJAU 4742 that was inserted into the commercial plasmid pMD19 *in prior* (7E721I3, Vazyme, China).

#### Global survey of *Trichoderma asperelloides* occurrences

Global occurrences of *T. asperelloides* were assessed using the barcode-verification procedure established for the genus [15]. We queried the NCBI Nucleotide database with BLASTn against the reference *tef1* and *rpb2* sequences of *T. asperelloides* X01119 and retained hits meeting the established similarity thresholds (*tef1* ≥ 97% or *rpb2* ≥ 99%, with ≥90% coverage). Because the species was formally described only in 2010 and is frequently deposited under other names, depositor-assigned taxonomic labels were disregarded, and records were curated based on diagnostic DNA barcode similarity. Redundant entries belonging to the same strain were collapsed using strain codes, sequence identity, and depositor information. For each non-redundant record, substrate and geographic metadata were manually extracted from the GenBank submission.

To evaluate whether the coastal isolates recovered in this study represented a distinct haplogroup within *T. asperelloides*, we generated a global *tef1* haplotype network. Public *tef1* sequences assigned to, or matching, *T. asperelloides* based on Cai and Druzhinina [15] were retrieved from GenBank and combined with *tef1* sequences from all isolates recovered in this study, including 11 non-coastal strains. Representative reference taxa were added for context, including ex-type *T. asperellum* CBS 433.97, ex-type *Trichoderma yunnanense* CBS 121219, and ex-type *T. atroviride* IMI 206040. Sequences were aligned using MUSCLE [37] and manually curated in AliView. The alignment was trimmed to a shared *tef1* region of 460 nucleotide positions. Records with insufficient overlap or extensive missing data were excluded, yielding a final dataset of 415 sequences. A TCS haplotype network [43] was inferred in PopART v1.7 [44]. Identical sequences were collapsed into haplotypes, and node size was scaled according to the number of records represented by each haplotype. Geographic categories were assigned from GenBank source metadata and from the sampling records of this study, using the following groups: China coastal isolates from this study, other China, Asia outside China, America, Europe, Africa, Oceania, unknown, and reference taxa. Substrate categories were grouped as coastal soil, soil/rhizosphere non-coastal, plant-associated substrate, and not defined/other.

### Statistical analysis

All experiments, unless specified otherwise, were conducted with at least three biological replicates. The expression pattern of genes of interest at each time point was illustrated either by bar plot or heatmap, generated in R (v4.3.0). Outliers within the absolute qPCR data were identified and removed using the interquartile range (IQR) method within each treatment group and strain. Values falling below Q1–1.5 × IQR or above Q3 + 1.5 × IQR were considered outliers and excluded from subsequent statistical analyses. The results of NMT were shown with Raincloud plot [45] in R. *Trichoderma* community composition along the coastal transect was analyzed by Canonical Correspondence Analysis (CCA) in CCA package in R (https://cran.r-project.org/web/packages/CCA/index.html), using a site-by-taxon matrix of *Trichoderma* recovery data and measured soil variables, including EC, pH, total carbon, and total nitrogen, as explanatory variables; taxon scores were calculated to summarize the association of each recovered taxon with the sampled sites and environmental variables. One-way analysis of variance (ANOVA) and Tukey’s HSD multiple comparison analyses were performed in R to test whether the removal of target gene significantly affected the halotolerance on plates (*n* ≥ 3), the total fungal DNA in microcosm (absolute qPCR, *n* = 8). Student’s *t*-test was used for halotolerance, *gld1* expression, and glycerol production between the mutant and the wild type. The significance level was set at *P* < .05, unless otherwise stated.

## Results

### Coastal survey reveals limited *Trichoderma* diversity and frequent recovery of halotolerant *Trichoderma asperelloides*

**Figure 1:**
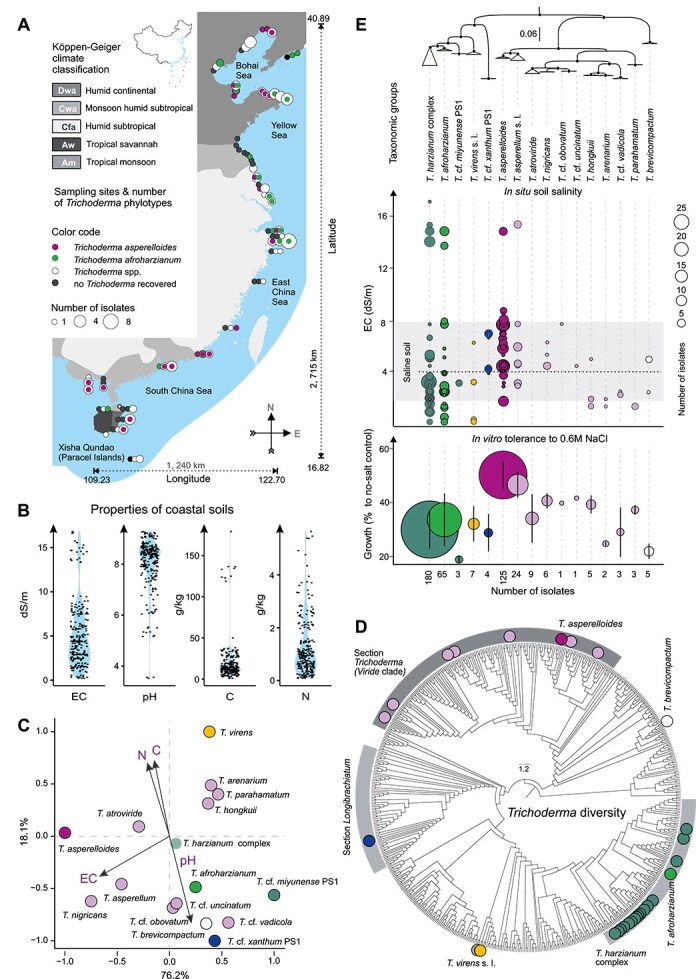
*Trichoderma* diversity and halotolerance along a coastal transect. (A) Map of 83 sampling sites spanning five major Köppen–Geiger climate zones (legend, top left), showing the distribution and diversity of *Trichoderma* phylotypes. Symbol size indicates the number of *Trichoderma* isolates recovered per site; color denotes dominant taxa: *T. asperelloides* (magenta), *T. afroharzianum* (green), other *Trichoderma* spp. (white), or no recovery (black). The base map was sourced from the Standard Map Service (GS(2019)1651) issued by the Ministry of Natural Resources of China. National boundaries remain unmodified. (B) Distribution of key soil chemical properties across sampling sites: electrical conductivity (EC, used as a proxy for salinity), pH, total organic carbon (C), and total nitrogen (N). (C) CCA of *Trichoderma* community composition constrained by measured soil variables. Arrows indicate EC, pH, total organic carbon, and total nitrogen. Points represent taxon scores; each *Trichoderma* taxon is shown as a summary of its association with the sites where it was recovered and the corresponding soil variables. (D) Genus-wide reference phylogeny of *Trichoderma* based on concatenated ITS, *tef1*, and *rpb2* sequences from ex-type strains for all species and other essential isolates (updated based on [15]). Major sections and species complexes are annotated, including the *Viride* Clade (*T. asperelloides*), *Longibrachiatum* Section, *Harzianum* species complex (*T. afroharzianum*), *T. virens*, and *T. brevicompactum*. (E) Taxon-level ecological and physiological responses to salinity. Top: simplified phylogeny of major groups recovered from coastal soils. Middle: *in situ* soil salinity (EC) for each taxon; shaded area denotes EC range typical of saline soil. Bottom: *in vitro* halotolerance (growth on 0.6 M NaCl, expressed as % of the control); circle size reflects the number of isolates sampled (middle) and tested per taxon (bottom).

To assess the ecological distribution and salinity tolerance of *Trichoderma* along the terrestrial–marine ecotone, we sampled 83 coastal sites spanning a 2700 km transect across the Bohai, Yellow, East China, and South China Seas. This transect crossed five Köppen–Geiger climate zones [46], from seasonally frozen, humid continental regions in the north to tropical monsoon and savannah climates on Hainan Island, with humid subtropical and monsoonal zones in between (Fig. 1A). Soil chemistry varied across sites, reflecting differences in tidal influence, vegetation, and anthropogenic disturbance. Total organic carbon and nitrogen were moderate, although most soils were slightly alkaline (mean pH 8.4; Fig. 1B). Electrical conductivity (EC), a proxy for salinity, ranged from 0.2 to 16 dS m^−1^, with lowest values in freshwater-influenced deltaic soils.

From 57 of 83 sites, we recovered 443 *Trichoderma* isolates. Cultivable diversity was represented by a few cosmopolitan taxa, notably *T. afroharzianum* and *T. asperelloides*, which spanned the entire transect but displayed distinct ecological profiles (Fig. 1C). The *Viride* clade was among the most represented, primarily due to *T. asperelloides*, whereas other members of this clade were rare (Fig. 1D and E). The *T. harzianum* species complex, including *T. afroharzianum*, was the most frequent overall. Within this complex, we identified at least 16 putatively novel, geographically scattered phylotypes, none were dominant at any site, suggesting localized, under-sampled diversity (Table 1). Additional isolates belonged to *T. virens, T. brevicompactum*, and section *Longibrachiatum* (Fig. 1D).

Phylotype richness ranged from one to eight per site, with 37 of 57 sites harboring only one or two. Most taxa were associated with low EC soils, although *T. afroharzianum* displayed the broadest salinity range. In contrast, *T. asperelloides* was consistently linked to moderate and high *in situ* salinity (Fig. 1E, top). To assess intrinsic halotolerance, all isolates were tested in vitro for growth on 0.6 M NaCl. Cumulatively, *T. asperelloides* maintained reduced albeit substantial growth under these conditions, confirming its physiological tolerance to salt stress among the tested isolates (Fig. 1E, bottom), whereas most other taxa were essentially less resilient to the osmotic stress. To test whether this phenotype was restricted to the Chinese coastal isolate set, we additionally phenotyped representative *T. asperelloides* strains from non-coastal sources and reference materials included in the global *tef1* framework (Table 1). These strains retained substantial growth under NaCl stress, indicating that the salt-tolerant phenotype is not confined to coastal isolates from this study (Supplementary Fig. 1).

The coastal survey and growth assays identified *T. asperelloides* as the recovered taxon most consistently associated with saline sites and intrinsic halotolerance. We therefore selected strain X01119 as a representative model to dissect the genetic basis of salinity tolerance.

### Functional screening identifies an aldo-keto reductase required for halotolerance in *Trichoderma asperelloides* X01119.

**Figure 2:**
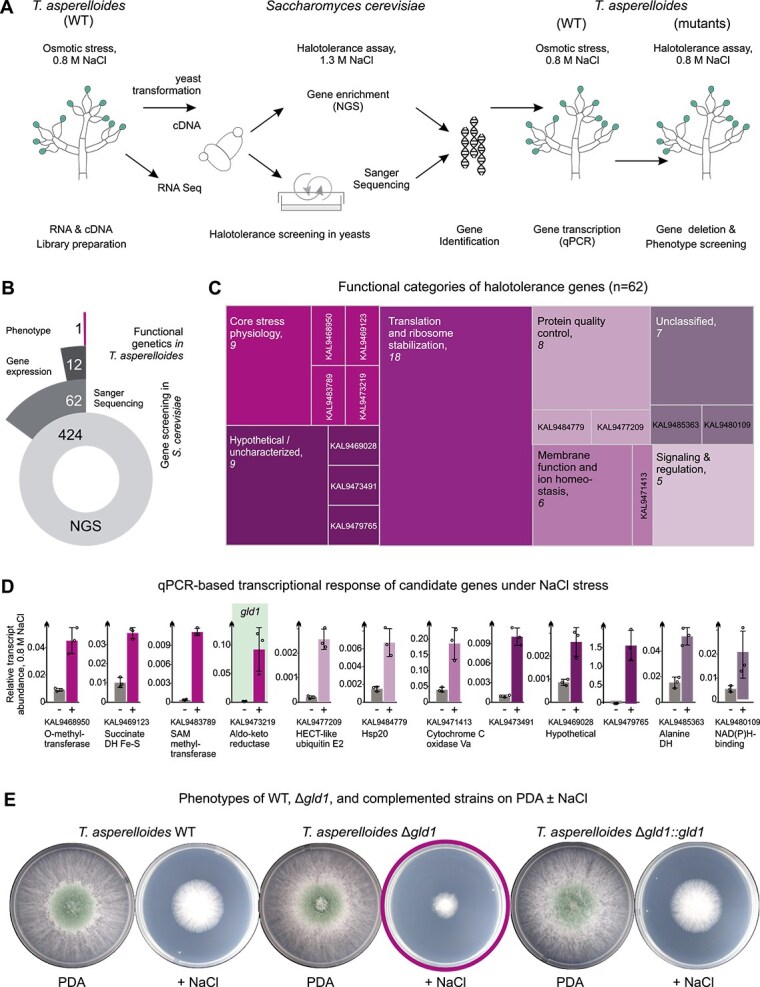
Functional screening identifies *gld1* as an essential gene for halotolerance in *T. asperelloides.* (A) Experimental workflow. A salt-induced cDNA library from *T. asperelloides* was constructed and transformed into *S. cerevisiae*. Transformants were screened for halotolerance (with 1.3 M NaCl), and enriched inserts were identified via NGS (Illumina HiSeq). Stable halotolerant colonies were isolated and sequenced by Sanger sequencing to identify individual *Trichoderma* genes. Candidate genes were assessed for transcriptional induction in *T. asperelloides* under osmotic stress (0.8 M NaCl) and functionally validated via gene deletion. (B) Sunburst plot summarizing gene discovery. A total of 424 *T. asperelloides* genes were found to be enriched in the salt-tolerant yeast population, as revealed by NGS (Supplementary Data 6). Of these, 61 were also identified from individual yeast clones that retained halotolerance after repeated subculturing, and 1 additional gene was included based on its strong enrichment in the NGS dataset, yielding 62 candidate genes. Twelve of these genes were transcriptionally induced in *T. asperelloides* under NaCl stress and selected for functional validation. Deletion of one gene (*gld1*, KAL9473219) resulted in a halotolerance-deficient phenotype. (C) Functional classification of the 62 *T. asperelloides* genes associated with halotolerance in yeast. (D) Transcriptional responses of the 12 salt-induced genes measured by qPCR under control (–) and osmotic stress (+) conditions in *T. asperelloides*. Only deletion of the aldo-keto reductase gene *gld1* (KAL9473219) impaired halotolerance (panel E). (E) Growth phenotypes of wild type (WT), Δ*gld1*, and the *gld1*-complemented strain (*Δgld1::gld1*) on PDA and PDA + 0.8 M NaCl. The Δ*gld1* mutant had strongly reduced growth under salt stress, confirming *gld1* is essential for halotolerance.

To dissect the genetic basis of halotolerance, we combined transcriptomics and yeast-based functional screening (Fig. 2A). A salt-induced cDNA library was constructed from *T. asperelloides* strain X01119 grown under osmotic stress (0.8 M NaCl), and transformed into *S. cerevisiae*. Following selection on 1.3 M NaCl, Illumina sequencing identified 424 *T. asperelloides* genes enriched in the halotolerance-gained yeast population (Fig. 2B, Supplementary Data 6). Repeated subculturing yielded 62 stable transformants on high salinity condition, which were individually Sanger sequenced and functionally annotated (Fig. 2C). These candidate genes included functions in translation and ribosome stabilization (18), core stress physiology (9), protein quality control (8), membrane function and homeostasis (6), and signalling/regulation (5), whereas 16 genes were uncharacterized or hypothetical (Supplementary Data 6). Of the 62 inserts identified from yeast-based screening, 52 represented unique genes, and 10 were chimeric. Among the unique genes, 28 ranked among the most enriched in the NGS dataset, 22 showed moderate to low enrichment, and one was entirely absent from the NGS pool (Fig. 2). We next assessed the transcriptional response of these 62 candidates in *T. asperelloides* under salt stress. qPCR revealed that only 12 genes were significantly upregulated on PDA + 0.8 M NaCl (foldchange >3; Fig. 2D, Supplementary Data 7). One additional gene (KAL9476955) was included in further gene deletion due to its top enrichment ranking in the NGS screen.

Gene deletion mutants were generated for each of these 13 genes and phenotyped for growth under osmotic stress (Table 1). Only one gene, identified as homologous to aldo-keto reductase *gld1* [19, 20] (see below), proved important for growth on the salt-amended medium: deletion of gene KAL9473219 caused a marked growth reduction on NaCl (Fig. 2E). Complementation with the wild-type allele fully restored halotolerance. Two of the mutants (Δ*KAL9471413* and Δ*KAL9476955*) failed to grow on PDA lacking the salt amendment and were therefore excluded from further analyses. These results identify *gld1*, as a significant factor of osmotic stress tolerance in *T. asperelloides* X01119.

### GLD1 is essential for halotolerance and reflects dynamic evolution of fungal aldo-keto reductases

**Figure 3:**
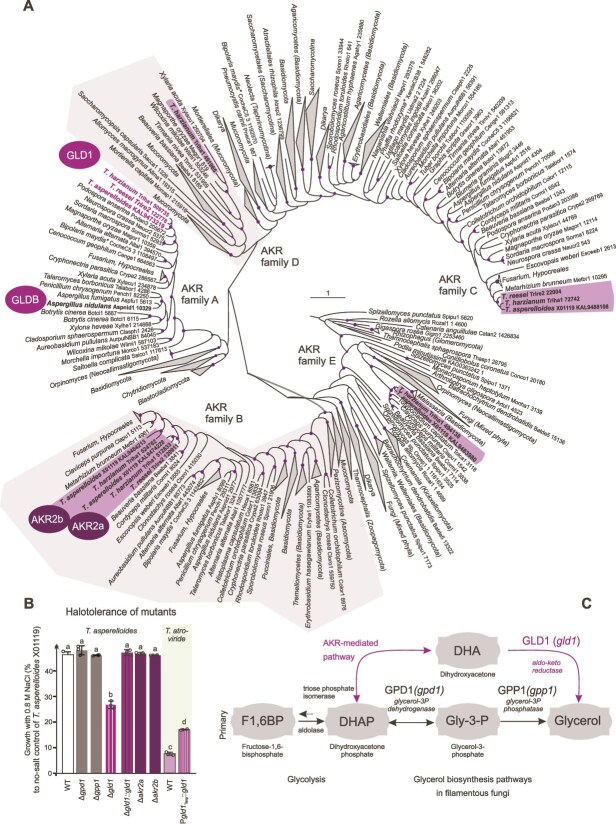
Phylogenetic and functional context of *Trichoderma* aldo-keto reductases (AKRs) in glycerol biosynthesis and halotolerance. (A) Maximum-likelihood phylogeny of fungal aldo-keto reductases (AKRs), resolving five major families (A–E). The GLD1 protein encoded by *T. asperelloides gld1* clusters within AKR family A together with experimentally characterized fungal GLD/GLDB proteins, including *A. nidulans* GLDB [19]. AKR family B contains *Trichoderma*-specific paralogs designated here as AKR2a and AKR2b. Families C–E contain AKR homologs of unknown function. *T. asperelloides* proteins are highlighted in purple; shaded areas indicate major AKR families; bootstrap values >70 are shown. (B) Growth of *akr* and *gld1* mutants on PDA supplemented with 0.8 M NaCl, shown as relative growth (%) compared with *T. asperelloides* X01119 (WT) on no-salt control (set to 100%). For *T. asperelloides*, only the Δ*gld1* mutant exhibited severely impaired halotolerance, confirming GLD1 as the key determinant of osmoprotection. In *T. atroviride*, replacement of the native P_Tatro_*gld1* promoter with the *T. asperelloides* promoter (P_Tasp_*gld1::gld1*) increased salt tolerance ≥2-fold relative to the wild type, demonstrating that regulatory differences in *gld1* expression are sufficient to confer enhanced halotolerance. Error bars represent SD (*n* = 3); letters indicate significant differences by Tukey’s HSD (*P* < .05). (C) Glycerol biosynthesis pathways in filamentous fungi. The canonical pathway (black) reduces dihydroxyacetone phosphate (DHAP) to glycerol via GPD1 encoded by *gpd1* and GPP1 encoded by *gpp1* through glycerol-3-phosphate. An AKR-mediated route (magenta) reduces dihydroxyacetone (DHA) to glycerol via GLD1 encoded by *gld1*.

To contextualize the function of GLD1 within the aldo-keto reductase (AKR) enzyme family, we mined 142 fungal genomes and retrieved 626 AKR homologs sharing ≥35% amino acid similarity with protein KAL9473219. Phylogenetic analysis resolved five major AKR clades (Fig. 3A). Protein encoded by KAL9473219 gene clustered within AKR family A alongside orthologs previously designated GLDB (*gldB*) in *A. nidulans* [19], GLD1 (*gld1*) in *T. atroviride* [20], and GLD2 (*gld2*) in *T. reesei* [21]. We therefore designated it GLD1. Notably, this clade also includes AKRs from saprotrophic sordariaceous fungi such as *Podospora, Sordaria*, and *Neurospora*. The topology deviates from the pattern expected under simple vertical inheritance in *Pezizomycotina*, suggesting a complex evolutionary history that may include lateral gene transfer (LGT), although this was not formally tested here. AKR family A was enriched among saprotrophs occupying nutrient-rich but ephemeral substrates (e.g. dung, litter, wood), many of which are also opportunists in disturbed soils.

AKR family B comprised *Trichoderma*-specific paralogs, here designated AKR2a and AKR2b, which were conserved in the *Harzianum* and *Viride* clades (Fig. 3A). *Trichoderma reesei* contained only one AKR family B homolog. This protein had previously been designated GLD1 [21], but it is distinct from GLD1 in *T. atroviride* [20] and from the GLD1/GLDB family analyzed here. AKR families C and E were broadly distributed across *Hypocreales*, although family E was absent from *T. reesei* and other members of section *Longibrachiatum*. The function of these proteins remains unknown. AKR family D is present exclusively in *T. harzianum* and clustered with AKRs from *Xylaria* and *Magnaporthe*, suggesting additional interlineage gene flow. Transcriptome data confirmed that all *Trichoderma* AKR genes are transcribed under at least one tested condition (Supplementary Data 7), ruling out pseudogenization. To evaluate possible adaptive evolution of *gld1*, we applied CodeML branch-site models to seven representative *Trichoderma* species (including five strains per *T. asperelloides, T. asperellum, T. atroviride, T. afroharzianum, T. harzianum, T. virens*, and *T. longibrachiatum*). These LRTs compared a model allowing a subset of sites to evolve under positive selection on the foreground branch (ω > 1) with the corresponding null model in which this class was constrained to neutral or conserved evolution (ω ≤1). In all cases, LRTs failed to reject the null hypothesis of purifying selection, with ω values well below 1 (Supplementary Data 3). This indicates that *gld1* is subject to strong evolutionary constraint across the tested lineages, consistent with its conserved role in osmoregulation. To test whether differences in promoter activity could account for species-level divergence in halotolerance, we examined *T. atroviride* IMI 206040, which showed the weakest salt tolerance within all of the tested strains (Fig. 1). Its native promoter (P_Tatro_*gld1*) was replaced by the *T. asperelloides* promoter (P_Tasp_*gld1*), yielding a promoter-swap strain of *T. atroviride* P_Tasp_*gld1::gld1*. When exposed to 0.4–0.8 M NaCl, the promoter-swap mutant exhibited at least a two-fold increase in halotolerance relative to the wild type (Fig. 3B), accompanied by an approximately 1.8-fold rise in *gld1* transcript abundance (Supplementary Fig. 2). These data indicate that promoter divergence can enhance osmotic fitness and support regulatory adaptation as a basis of the halotolerant phenotype.

We further compared the 1.5-kb upstream region of *gld1* across representative *T. asperelloides* strains spanning coastal and non-coastal sources. The promoter architecture, including the motifs identified by STREME, was conserved among the tested *T. asperelloides* representatives, including strains from low-salt/non-coastal substrates (Supplementary Fig. 2; Supplementary Data 4). These motifs are therefore not unique to the strain X01119, although their functional contribution to *gld1* regulation will require targeted promoter analysis.

Functionally, GLD1 contributes to glycerol biosynthesis via an accessory AKR-mediated route, distinct from the canonical GPD1/GPP1 pathway (Fig. 3C, Supplementary Fig. 3). Exogenous glycerol partially rescued growth at low concentrations, and enzymatic assays verified NADP^+^-dependent conversion of dihydroxyacetone to glycerol in the wild type but not in Δ*gld1* (Supplementary Figs 3 and 4). Additionally, ion flux measurements revealed sustained Na^+^ influx in Δ*gld1* hyphae under salt stress, consistent with compromised osmotic homeostasis (Supplementary Fig. 4). Deletion of glycerol-biosynthesis related genes showed that only *gld1* was essential for halotolerance. Mutants lacking *gld1* exhibited severely reduced growth under salt stress, whereas deletion of *gpd1, gpp1, akr2a*, or *akr2b* had no observable impact (Fig. 3B). Consistently, only *gld1* exhibited strong and specific induction (up to 300-fold) in response to osmotic and ionic stress, whereas *akr2a* responded exclusively to H₂O₂ and *akr2b* showed moderate upregulation (6–10-fold) across all stressors (Supplementary Fig. 5). Deletion of *tmk3*, which encodes the terminal MAP kinase of the high-osmolarity glycerol (HOG) pathway [47] and is orthologous to *HOG1* in *S. cerevisiae*, and *sakA* in *A. nidulans* [48], produced a halosensitive phenotype that could only tolerate 0.2 M NaCl and did not grow at higher concentrations of salt (Supplementary Fig. 5C). This result is consistent with the assumption that *gld1* functions downstream of the HOG signalling cascade. This is further supported by the strong transcriptional induction of *gld1* by NaCl, KCl, and sorbitol, but not H₂O₂, mirroring the known specificity of the HOG pathway. Consistently, *gld1* induction was abolished in the Δ*hog1* mutant, confirming that its activation depends on HOG signalling (Supplementary Fig. 5D).

Because coastal salinity fluctuates, we also examined whether *gld1* regulation differed during osmotic shock. Most *Trichoderma* species displayed a transient *gld1* transcriptional peak within ~2 h after NaCl exposure, whereas *T. asperelloides* maintained induction for up to 8 h (Supplementary Fig. 6). Under prolonged osmotic stress, all tested strains accumulated glycerol, but only *T. asperelloides* X01119 combined glycerol accumulation with sustained growth (Supplementary Fig. 7). These results indicate that halotolerance in *T. asperelloides* reflects not only glycerol production but also sustained regulatory activation and growth under salinity.

These findings establish GLD1 as a critical component of an accessory glycerol biosynthesis pathway required for halotolerance in *T. asperelloides* X01119, and highlight how AKR gene family diversification may have facilitated fungal adaptation to transient and saline habitats.

### Synthetic microcosm assay identifies *gld1* as required for *Trichoderma asperelloides* fitness under salinity stress

To assess the ecological importance of *gld1*, we developed a synthetic microcosm using gamma-irradiated soil (Entisols) previously characterized for *Trichoderma* colonization [9]. Salinity was adjusted to 0.2 M and 0.4 M NaCl to simulate moderate coastal conditions; no-salt soil served as the control. This design enabled direct evaluation of *T. asperelloides* fitness within a multispecies context under defined saline soil-like conditions.

Because ITS cannot resolve closely related *Trichoderma* phylotypes [15], we used a customized amplicon-sequencing strategy targeting a species-informative fragment of the single-copy *rpb2* gene encoding RNA polymerase II second-largest subunit (Fig. 1D, Table 1).

**Figure 4:**
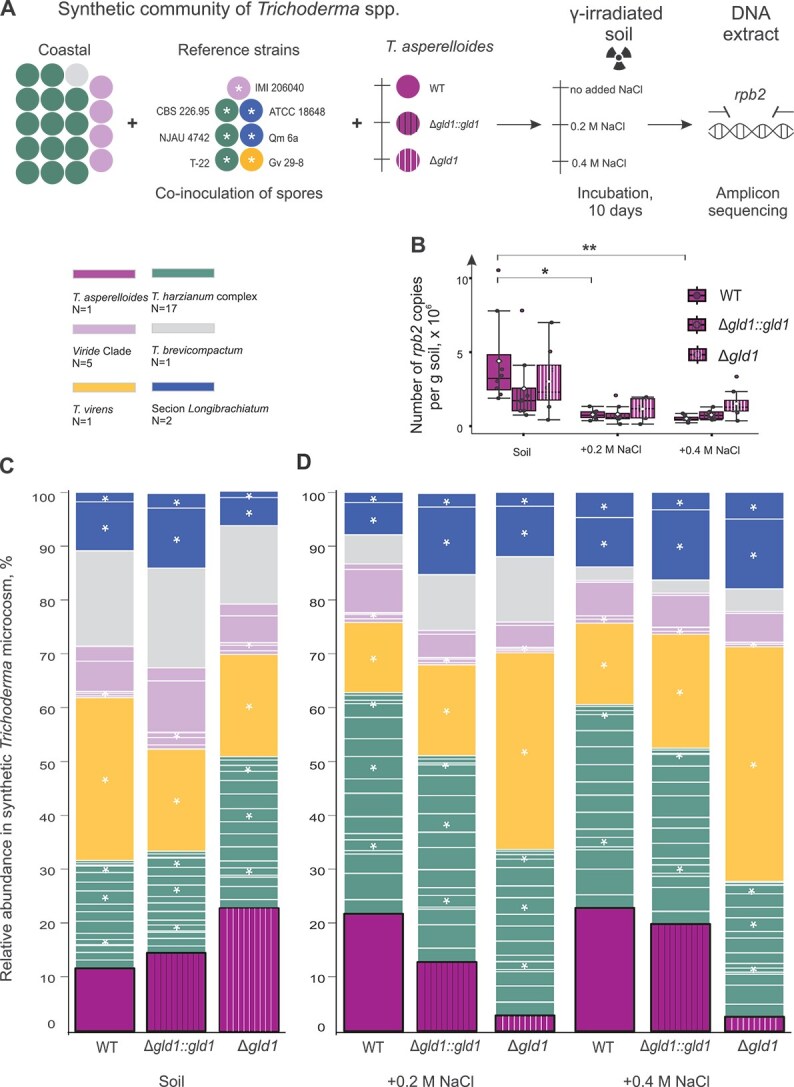
The *gld1* gene determines competitive fitness of *T. asperelloides* in saline soil microcosm. (A) Design of synthetic community microcosm experiment. A total of 29 *Trichoderma* strains (27 species) were combined, including 20 coastal isolates (this study), seven reference strains widely used in agriculture and biotechnology: *T. harzianum* CBS 226.95, *T.* sp. NJAU 4742, *T. afroharzianum* T-22, *T. atroviride* IMI 206040, *T. virens* Gv 29-8, *T. reesei* QM 6a, *T. longibrachiatum* ATCC 18648, and *T. asperelloides* X01119 (wild type, Δ*gld1*, or Δ*gld1*::*gld1*) as a predictor. The soil matrix was gamma-irradiated forest soil with three NaCl levels (0, 0.2 M, and 0.4 M). After 10 days of incubation, fungal community composition was assessed by *rpb2* amplicon sequencing and total DNA quantification. (B) Absolute quantification of total *Trichoderma* DNA in soil microcosms by qPCR targeting *rpb2*. Fungal biomass declined significantly under saline conditions compared to the non-saline control (Student’s *t*-test, *, *P* < .05; **, *P* < .01; ***, *P* < .001), but no statistically significant differences were observed between 0.2 M and 0.4 M NaCl. (C, D) Relative abundance of *Trichoderma* species in non-saline (C) and saline (0.4 M NaCl) (D) microcosms based on *rpb2* amplicon sequencing. Reference strains are indicated with asterisks. Due to large differences in total fungal DNA between saline and non-saline conditions (panel B), relative abundance results are presented separately. In non-saline conditions (C), *T. virens, T. brevicompactum*, and *T. asperelloides* wild type were among the dominant taxa. In saline microcosms (D), wild-type *T. asperelloides* dominated, whereas the Δ*gld1* mutant was displaced by other species (such as *T. virens*), and the complemented strain restored competitiveness.

The synthetic community was comprised of conidia from 20 coastal isolates together with 7 widely used reference *Trichoderma* strains (Fig. 4A). To test the role of *gld1*, we introduced either wild-type *T. asperelloides* X01119, its Δ*gld1* mutant, or the complemented strain (Δ*gld1*::*gld1*) into the community. After 10 days, amplicon sequencing of *rpb2* was used to profile community composition.

qPCR quantification of *Trichoderma* DNA per gram of soil revealed a strong effect of NaCl concentration on total *Trichoderma* DNA abundance (two-way ANOVA, *P* < .001), whereas *T. asperelloides* genotype had no significant effect (Fig. 4B). Tukey’s HSD confirmed significant differences in total *Trichoderma* DNA between salt and control conditions (*P* < .05), with no significant interaction between NaCl and *T. asperelloides* strains. Because qPCR and amplicon sequencing quantify DNA rather than transcriptional activity, these results were interpreted as endpoint community profiles.

Under non-saline conditions, the endpoint community profile was dominated by *T. virens, T. brevicompactum*, and *T. asperelloides*, with *T. reesei* contributing moderately (Fig. 4C). Δ*gld1* showed a modest relative increase compared to that of the WT in these conditions, possibly due to lower metabolic cost in the absence of stress. In contrast, under saline conditions, wild-type *T. asperelloides* X01119 became the dominant member. This competitive advantage was lost in the Δ*gld1* mutant, which was displaced by *T. virens*. Complementation restored salinity-dependent dominance (Fig. 4D) regardless of NaCl concentration.

The *rpb2*-based community readout was validated *in silico* and experimentally. Simulated reads with up to eight substitutions were reassigned correctly in >99.8% of cases, and TaqMan probe qPCR profiles for eight representative community members were consistent with the amplicon data (Supplementary Fig. 8, Supplementary Data 5).

Together, our results identify *gld1* as a functional determinant of *T. asperelloides* X01119 fitness under salinity stress and support its role in persistence under saline soil-like conditions.

### Global records indicate recurrent soil/rhizosphere recovery of *Trichoderma asperelloides*

**Figure 5:**
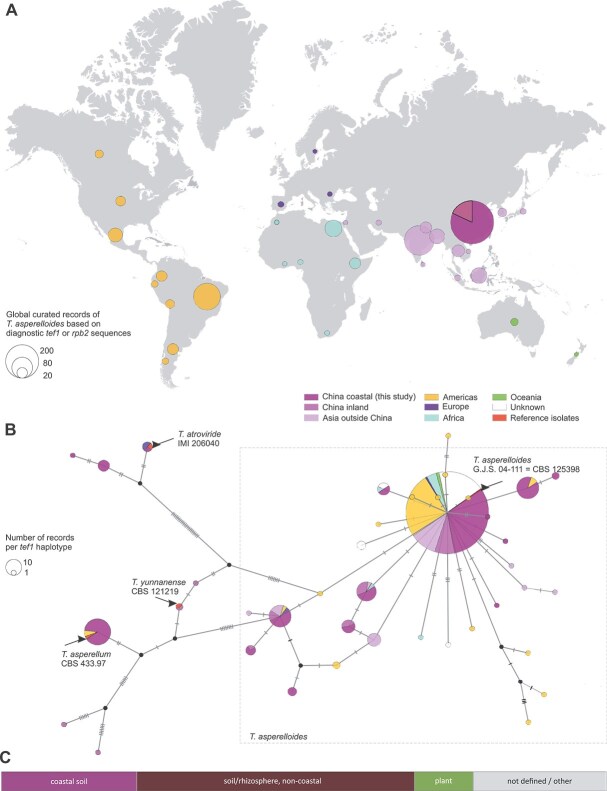
Global distribution, *tef1* haplotype structure, and source metadata of *T. asperelloides.* Occurrence records were compiled from public databases based on diagnostic sequence similarity to *T. asperelloides* (*rpb2* ≥ 99%, *tef1* ≥ 97%) and combined with isolates recovered in this study (Table 1). (A) Global biogeographic distribution of curated *T. asperelloides* records. Circle size is proportional to the number of records per country; colors indicate broad geographic categories. (B) TCS haplotype network inferred from a 460-position shared diagnostic *tef1* intron alignment comprising 415 sequences, including curated *T. asperelloides* records and close reference taxa. Node size is proportional to the number of records per *tef1* haplotype; colors follow panel A. Tick marks on branches indicate mutational steps. The dotted rectangle marks the *T. asperelloides* haplotype space. Coastal isolates from this study are embedded within this broader *T. asperelloides* diversity and do not form a distinct coastal haplogroup. (C) Substrate and habitat categories associated with curated *T. asperelloides* records from A. Coastal soils from this study account for 125 records, while other dominant sources include soil and rhizosphere environments. Plant-associated records and undefined or other sources are less frequent.

To place the coastal isolates in a broader distributional context, we mined public sequence databases using stringent identity thresholds of the diagnostic DNA barcodes (*tef1* ≥ 97% and *rpb2* ≥ 99%) [15]. Taxonomic labels were disregarded because *T. asperelloides* is a cryptic species within the *T. asperellum* complex. It was formally described in 2010 [23], and has frequently been deposited as *T. asperellum* or related taxa. Redundant entries were collapsed by strain, yielding 568 non-redundant global records, including 125 isolates from this study (Fig. 5).

Curated records indicate a broad, near-cosmopolitan distribution, with the largest numbers from China, India, Brazil, Egypt, Malaysia, Bangladesh, and Mexico (Fig. 5A). The curated distribution was concentrated mainly in tropical, subtropical, and warm-temperate regions, although the available records are strongly influenced by sampling intensity and sequence deposition patterns. Confirmed European records were rare in the curated dataset, represented mainly by Romania, Sweden, and the Canary Islands.

To test whether the coastal isolates recovered in this study represented a distinct Chinese coastal haplogroup, we generated a TCS haplotype network from a 460-position shared *tef1* hypervariable intron-containing alignment comprising 415 sequences, including public records, voucher sequences, and close reference taxa. The coastal isolates from this study were embedded within the broader *T. asperelloides* haplotype space, and no separate coastal haplogroup was resolved (Fig. 5B). This indicates that the coastal isolates do not represent a separate *tef1*-defined coastal lineage within *T. asperelloides*.

Source metadata showed that the largest category of curated *T. asperelloides* records originated from soil or rhizosphere environments (*n* = 261), followed by coastal soils from this study (*n* = 125), plant-associated substrates (*n* = 55), and records with undefined or other sources (Fig. 5C). Records from fungi, wood, marine habitats, clinical material, caves, and other rare sources were uncommon. These patterns support recurrent recovery of *T. asperelloides* from edaphic habitats, especially soils and rhizospheres, despite sampling bias. GlobalFungi provides consistent ITS-based context for the *T. asperellum* complex, but limited resolution for cryptic species [49].

The global metadata and *tef1* haplotype network indicate that the coastal isolates analyzed here fall within broader *T. asperelloides* diversity and that soil/rhizosphere-associated records dominate the curated public dataset. We therefore interpret the coastal recovery and halotolerance phenotype as recurrent among the tested *T. asperelloides* representatives, with strain X01119 providing the experimentally tractable model for mechanistic analysis.

## Discussion

Attributing fixed lifestyles to fungal species—or even higher taxonomic ranks—is a common practice in environmental and evolutionary microbiology, particularly in postgenomic studies where such groupings are used to infer or predict genetic traits [1]. However, these efforts are frequently undermined by the inherent ecological plasticity, widespread environmental opportunism, and functional pleiotropy of fungal life histories [50]. A single species may alternate between saprotrophic and biotrophic modes, shift between copiotrophic and oligotrophic growth, interact with multiple partners, and persist under fluctuating or extreme environmental conditions [18, 51]. Multi-omic studies have revealed not only extensive metabolic redundancy but also the key role of context-dependent accessory genes—typically residing in the shell genome and often acquired via LGT—in enabling this flexibility [9].

In this study, we questioned the ecological limits of a genetically compact and well-defined genus of sylvan, mycoparasitic, and wood-associated fungi [8–10], some of which are used in agriculture for plant protection and growth promotion [8, 13, 52]. Along a 2700 km coastal transect, we recovered a limited but consistent *Trichoderma* diversity, with saline soils frequently yielding *T. asperelloides* among cultured isolates [23]. This aligns with earlier observations that records from salt-affected and marine/coastal substrates are uncommon in a genus typically associated with sylvan and plant-linked habitats [9, 10, 17], though several marine *Trichoderma* isolates were reported [53]. Functional screening and genetic analysis identified *gld1*, encoding an aldo-keto reductase (AKR), as an accessory gene required for halotolerance in *T. asperelloides*. Although *gld1* orthologs are broadly distributed across *Trichoderma* genomes, their role in osmotic fitness appears amplified in *T. asperelloides* X01119, likely through regulatory adaptation. Deletion of *gld1* impaired both salt tolerance and competitive fitness in microcosms, supporting its role under saline soil-like conditions. Most entomopathogenic *Hypocreales* lack *gld1*, instead encoding AKR family B members, which are also present and duplicated in some *Trichoderma* species (e.g. *akr2a, akr2b*), but proved non-essential for halotolerance in our system. These findings raise the possibility that *Trichoderma* AKR family A genes were shaped by LGT and lineage-specific retention or loss to expand glycerol biosynthesis capacity—a hypothesis warranting further evolutionary investigation as a mechanism of ecological plasticity.

Our findings extend previous work on osmoadaptive AKRs such as *gldB* in *A. nidulans* [19] and *gld1* in *T. atroviride* [20], both linked to osmotolerance in submerged *in vitro* cultures. However, those studies relied on controlled laboratory assays. Here, *gld1* was discovered through an ecologically structured survey and functionally validated in soil microcosms containing diverse *Trichoderma* species, linking gene function to competitive fitness under salinity in a structured soil microcosm.

Yeast-based functional screening proved particularly effective, as it allowed the use of nutrient-rich PDA rather than minimal media typically employed to suppress background metabolism [54]. The high nutrition was instrumental for detecting *gld1* function, likely due to the high energetic demands of NADPH-dependent glycerol biosynthesis. The resulting phenotypes are consistent with nutrient-enriched soil-like conditions, highlighting the ecological relevance of *gld1*-mediated halotolerance and the importance of testing gene function under realistic nutrient regimes.

Our synthesis of global occurrence data supports recurrent recovery of *T. asperelloides* from soil-associated substrates, including cultivated soils and rhizospheres, while remaining compatible with broader opportunistic ecology and sampling bias. This pattern contrasts with the broader saprotrophic and mycotrophic versatility observed in other *Trichoderma* species [8, 17] and aligns with our finding that halotolerance can support persistence under saline soil-like conditions. The well-known biocontrol strain T203—originally described as *T. harzianum* and *T. asperellum* [55, 56], and later reclassified as *T. asperelloides*—has been studied for over two decades through functional genetics and molecular ecology [55–58]. Though often framed in applied contexts, this body of work yielded mechanistic insights into genes underlying plant-beneficial traits. These findings, together with our experimental data, position *T. asperelloides* as a tractable model for linking gene function to niche specialization. Although our metadata analysis did not reveal *in situ* associations of *T. asperelloides* with other fungi likely due to the limited sample size, its aggressive mycoparasitism is well documented *in vitro*—particularly for strain T203—and underpins its successful commercial use in sustainable agriculture [52, 55, 57, 59]. Thus, *T. asperelloides* combines experimentally supported salinity tolerance with a history of agricultural use, whereas its competitive performance in soil communities warrants biosafety assessment of unintended persistence in managed environments. The specific regulatory pattern of *gld1* may serve as a functional marker for selecting strains adapted to osmotic stress.

The breadth of the halotolerance phenotype should be interpreted in the taxonomic context of the *T. asperellum* species complex. *T. asperelloides* was separated from *T. asperellum sensu lato* as a cryptic sister lineage [23], and many public records remain deposited under earlier or incorrect names. The original species treatment also reported low intraspecific diversity, no known teleomorph, and an apparently clonal structure. Although this does not justify treating halotolerance as an invariant diagnostic character, it makes recurrent phenotypic conservation across geographically and ecologically distinct representatives biologically plausible. Our coastal dataset spans 2700 km and five climate zones, and the global *tef1* haplotype network did not resolve these isolates as a distinct coastal haplogroup. Additional non-coastal representatives also retained substantial growth under NaCl stress, and the *gld1* promoter architecture was conserved across the tested representatives. Together with published salinity-relevant observations on *T. asperelloides* strains, including Israeli strain T203 [53, 58] and plant-inoculation studies under saline conditions [60], these data support halotolerance as recurrent among the tested *T. asperelloides* representatives. The halotolerance observed in *T. asperellum* further suggests that the regulatory basis of this phenotype may predate the divergence of the broadly sympatric sister species *T. asperellum and T. asperelloides*, making this species pair an important target for future studies of regulatory evolution in *Trichoderma*.

GLD1 operates within an accessory glycerol biosynthesis pathway that complements the canonical GPD–GPP route. Deletion of *gld1* reduced glycerol accumulation and salt tolerance despite intact primary metabolism, and sustained *gld1* expression under prolonged osmotic stress indicates regulatory adaptation. These findings align with the adaptive pangenome model, in which accessory genes are maintained due to context-dependent fitness advantages [61]. Consistent with this, CodeML analyses across seven *Trichoderma* species revealed strong purifying selection acting on *gld1*, with no signal of positive selection. This suggests that the observed differences in halotolerance arise primarily from regulatory control, with little support for coding-sequence adaptation in this dataset. Consistently, the *T. atroviride* promoter-swap mutant (P_Tasp_*gld1::gld1*) acquired at least a two-fold improvement in halotolerance accompanied by a proportional increase in *gld1* transcription, directly linking promoter activity to osmotic fitness. Although a detailed evolutionary analysis of fungal AKRs is beyond the scope of this study, our phylogeny suggests at least five major AKR families in *Trichoderma*, with *gld1* clustering in family A. This clade is enriched in sordariaceous fungi inhabiting ephemeral, nutrient-rich, and stress-prone substrates such as dung and decaying wood.

These results reinforce the view that many so-called cryptic fungal species can exhibit clear ecological and physiological differentiation when examined using functional and environmental genomics [62]. Our results support ecological and physiological differentiation of *T. asperelloides*, a cryptic lineage within the *T. asperellum* complex [23]. This underscores the value of integrating ecological and physiological traits with morphology and DNA barcodes for species delimitation and functional profiling in fungi.

Our study also introduces a high-resolution amplicon sequencing strategy based on *rpb2*, which reliably differentiates *Trichoderma* diversity at the species level, including those unresolved by ITS [15, 23, 49, 63, 64]. This approach may prove broadly useful for diagnostics, biodiversity surveys, and regulatory compliance in other taxonomically complex fungal genera.

Together, our findings show how an accessory gene can contribute to ecological specialization in fungi, consistent with adaptive pangenome theory. By linking gene function to competitive fitness in structured communities, we provide a tractable model for understanding fungal opportunism and stress adaptation. The *gld1* gene exemplifies how conditionally essential pathways underpin fungal persistence in saline environments and contribute to ecological niche expansion.

## Supplementary Material

Supplementary_material_wrag162

## Data Availability

Raw sequence data, including *rpb2* amplicon sequencing, cDNA library reads, and whole-genome and RNA-seq data of *T. asperelloides* X01119, have been deposited in NCBI under BioProject accession numbers PRJNA1372685, PRJNA1372926, PRJNA1293129, and PRJNA1373383, respectively. The final genome assembly and annotations of *T. asperelloides* are available via GCA_052084505. The DNA barcoding sequences of the ITS, *tef1* and *rpb2* from *Trichoderma* isolates have been submitted to the NCBI GenBank database that can be retrieved using the accession IDs listed in Supplementary Data 1. Additional supporting data are provided as Supplementary Data. Further materials are available from the corresponding authors upon reasonable request.

## Materials availability

Strains of *Trichoderma* used in this study are available from F.M.C.
