# Supplementary material for: Regulatory adaptation of an accessory gene controls fungal halotolerance and niche expansion
Source: ISME J. 2026 Jun 26;20(1):wrag162. doi: 10.1093/ismejo/wrag162 (PMC13452454; doi:10.1093/ismejo/wrag162)
Supplement: Supplementary file 1 [file supplementary_material_wrag162.zip › Ding et al revision SI Figures.pdf]

Regulatory adaptation of an accessory gene controls fungal halotolerance and niche expansion

Mingyue Ding[1§], Guan Pang[2§], Mengting Huang[1], Yujie Shi[1], Fan Yi[3], Paolo Wong[3], Dongxin Shi[3], Zhilin Yuan[4], Lanxi Su[5], Junjie Guo[6], Weixiang Wang[7], Oded Yarden[8], Feng M. Cai[3]✉, Irina S. Druzhinina[9]✉, Qirong Shen[1]✉

[1] Jiangsu Provincial Key Lab of Solid Organic Waste Utilization, Key lab of organic-based fertilizers of China, Jiangsu Collaborative Innovation Center of Solid Organic Wastes, Educational Ministry Engineering Center of Resource-saving fertilizers, Nanjing Agricultural University, Nanjing, China

[2] College of Horticulture, Nanjing Agricultural University, Nanjing, China

[3] State Key Laboratory of Biocontrol, School of Ecology, Shenzhen Campus of Sun Yat-sen University, Sun Yat-sen University, Shenzhen, China

[4] Research Institute of Subtropical Forestry, Chinese Academy of Forestry, Hangzhou, China

[5] Spice and Beverage Research Institute, Chinese Academy of Tropical Agricultural Sciences, Wanning, China

[6] State Key Laboratory of Biocontrol, Guangdong Provincial Key Laboratory of Plant Stress Biology, School of Agriculture and Biotechnology, Shenzhen Campus of Sun Yat-sen University, Sun Yat-sen University, Shenzhen, China

[7] Beijing Key Laboratory of New Technology in Agricultural Application, Beijing University of Agriculture, Beijing, China

[8] Department of Plant Pathology and Microbiology, the Robert H. Smith Faculty of Agriculture, Food and Environment, The Hebrew University of Jerusalem, Rehovot, Israel

[9] Royal Botanic Gardens, Kew, Richmond, United Kingdom

[§] These authors contributed equally to this work

✉ Corresponding authors: Feng M. Cai, caif8@mail.sysu.edu.cn; Irina S. Druzhinina, i.druzhinina@kew.org; Qirong Shen, shenqirong@njau.edu.cn

Supplementary Figures

Supplementary Figure 1 | Halotolerance of coastal and non-coastal *Trichoderma* representatives

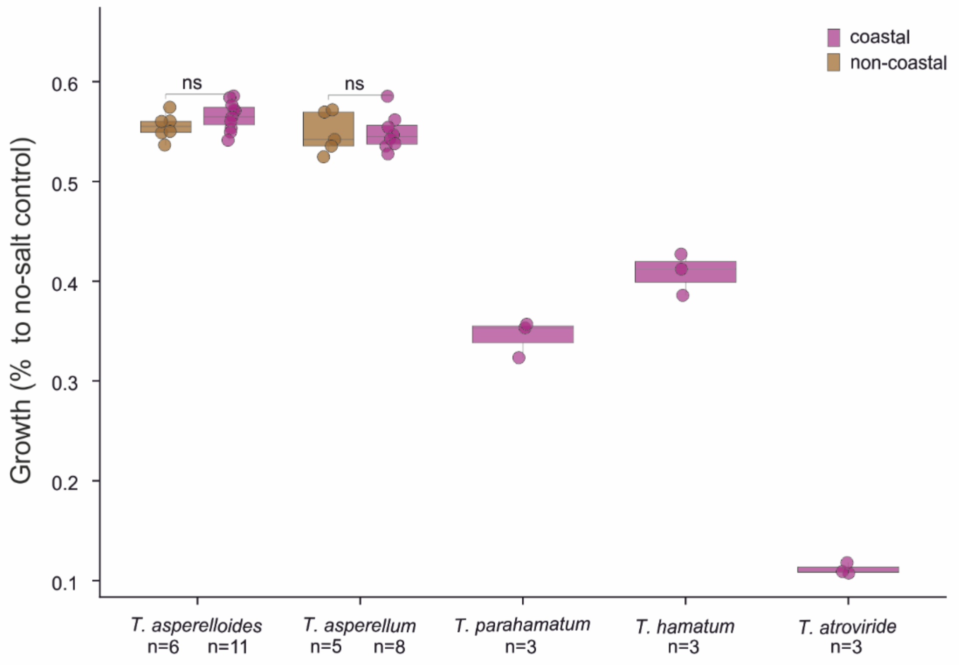

Relative growth on PDA supplemented with 0.6 M NaCl, expressed relative to growth on no-salt control medium. Coastal and non-coastal representatives of *T. asperelloides* and *T. asperellum* showed no significant difference in halotolerance, while additional *Trichoderma* taxa are shown for comparison. Points represent individual strains; boxes show the distribution within each group. ns, not significant, Student's t test at $P = 0.05$.

Supplementary Figure 2 | Promoter swap confirms the regulatory adaptation of *gld1* to osmotic stress

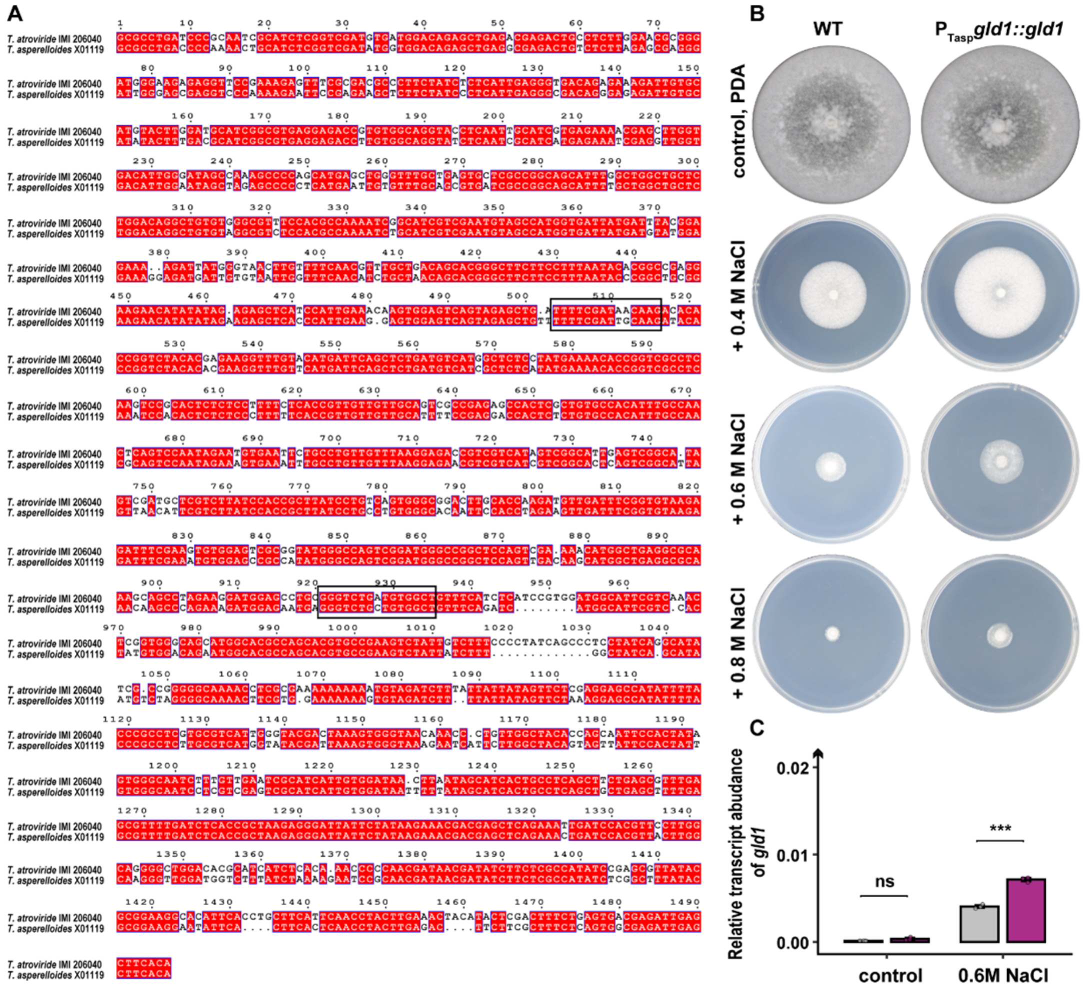

A, An alignment of ca. 1.5 kb promoter regions of *T. atroviride* IMI 206040 and *T. asperelloides* X01119. Enriched motifs (estimated by STREME [41]) in *T. asperelloides* relative to that of *T. atroviride* were highlighted with frames. **B**, Growth assays of the promoter-swap strain on PDA supplemented with 0.4-0.8 M NaCl. The promoter from *T. asperelloides* X01119 enables the NaCl-sensitive strain *T. atroviride* IMI 206040 being halotolerant (P_{Tasp}*gld1::gld1*, see quantitative data in **Fig. 3B**). **C**, The *gld1* transcriptional level in promoter-swap strain (P_{Tasp}*gld1::gld1*) significantly increased up to 1.8 fold compared to that of the WT strain (*T. atroviride* IMI 206040), in the presence of 0.6 M NaCl. Expression was measured by RT-qPCR, normalized to the reference gene (*tef1*), and averaged over biological replicates (n=3). Student's t test, ***, $P < 0.001$; ns, not significant at $P = 0.05$.

Supplementary Figure 3 | GLD1 is a NADPH-dependent DHA reductase involved in glycerol biosynthesis

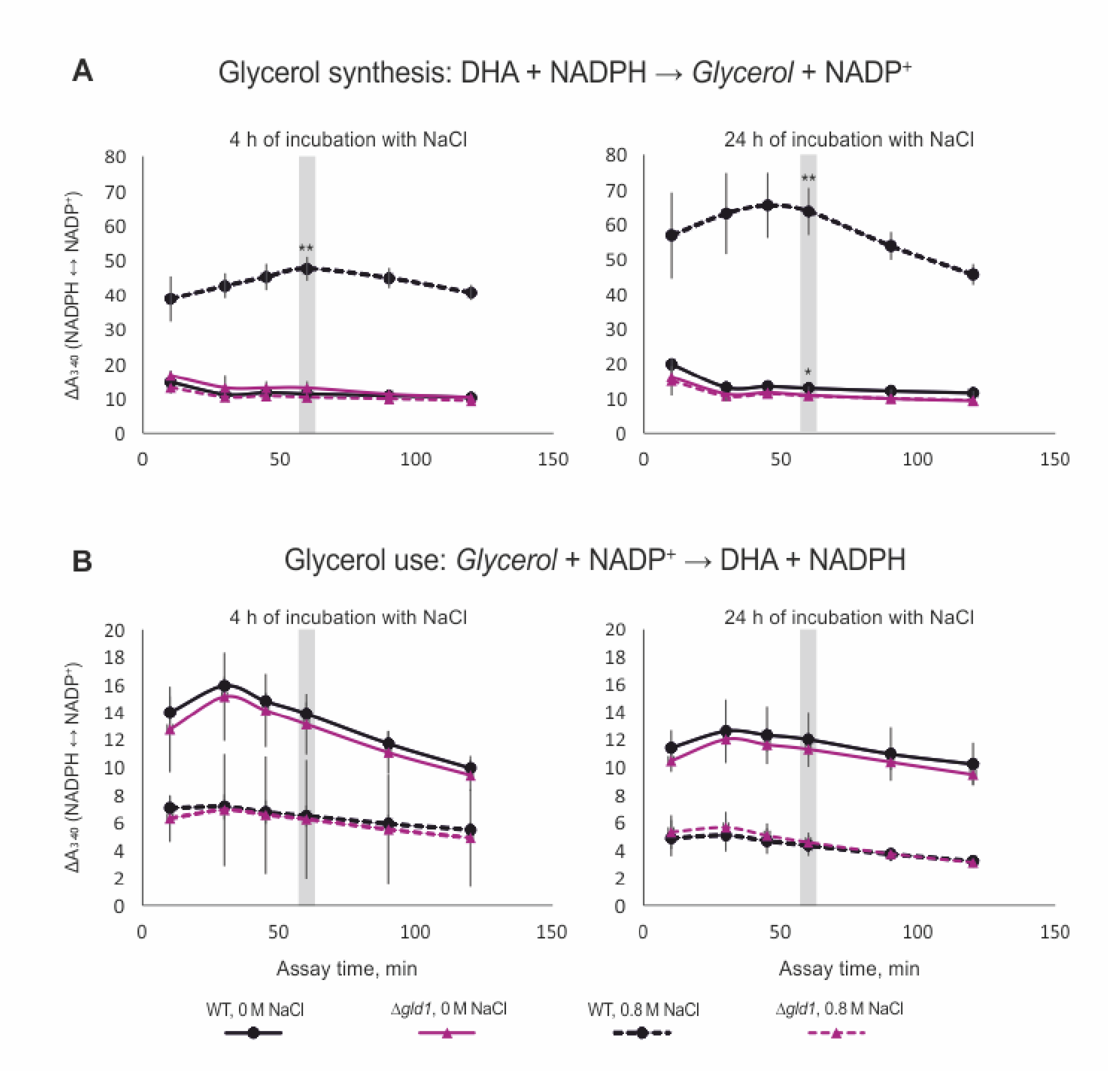

A, Enzyme activity measured by NADPH oxidation (ΔA_{340}) in crude extracts from *T. asperelloides* wild-type (WT) and Δ*gld1* strains after 4 h and 24 h of incubation in the presence or absence of 0.8 M NaCl. Activity corresponds to the reduction of dihydroxyacetone (DHA) to glycerol. **B**, Enzyme activity measured by $NADP^+$ reduction (ΔA_{340}) in the same extracts using glycerol as substrate, reflecting glycerol dehydrogenase activity. Grey shading indicates the time point used for endpoint quantification (60 min). Data represent mean ± SD, *n* = 3 biological replicates. Statistically significant differences between Δ*gld1* and WT are indicated by stars, Student's t test, *, $P < 0.05$; **, $P < 0.01$; ***, $P < 0.001$.

Supplementary Figure 4 | GLD1-mediated glycerol biosynthesis enables osmotic regulation of sodium influx in saline environments

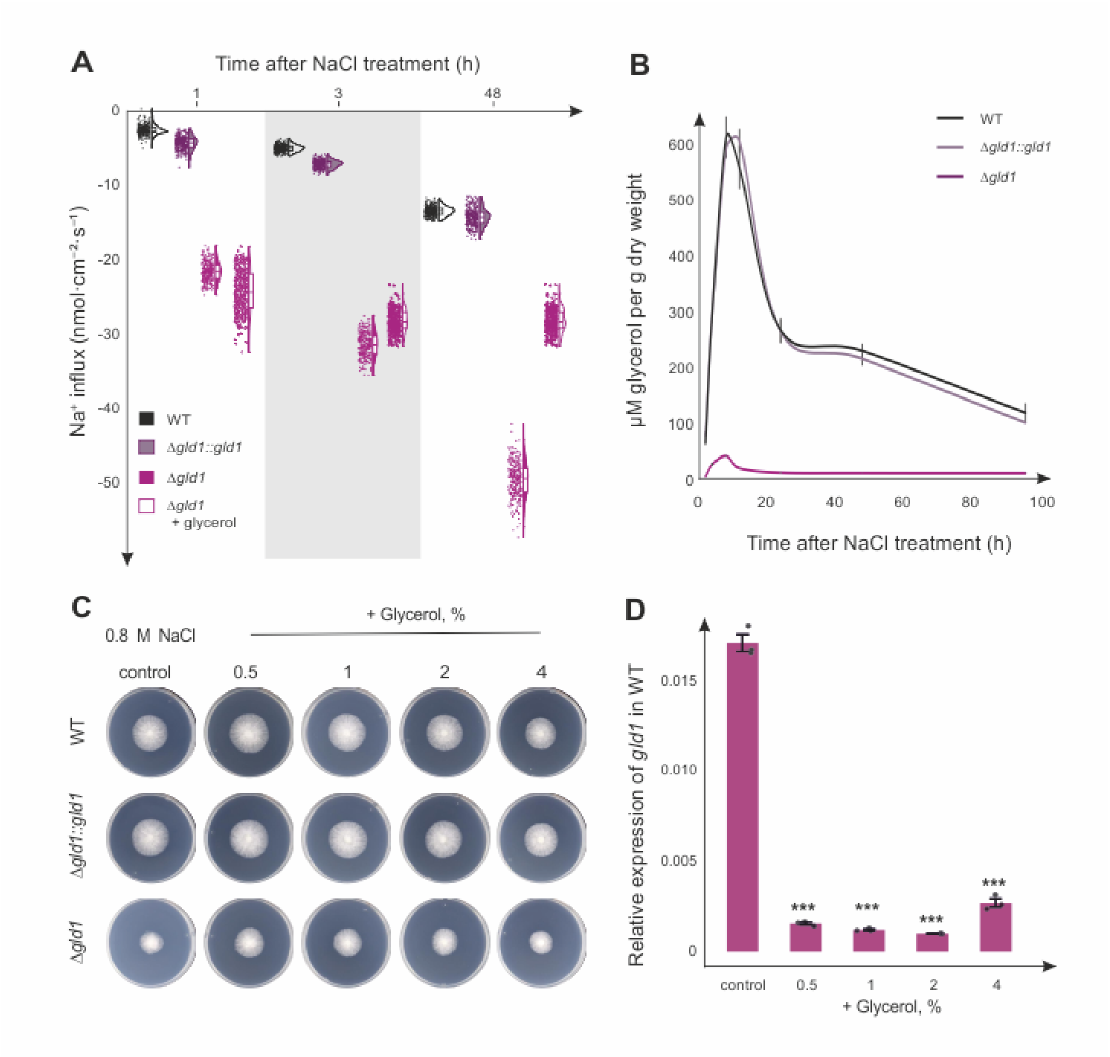

A, Net Na^{+} flux measured in surface-grown hyphae at 1, 3, and 48 h after 0.8 M NaCl treatment. Fluxes were measured by non-invasive microelectrode ion flux estimation (MIFE) and are expressed as nanomoles per square centimetre per second (fmol·cm^{-2}·s^{-1}). The Δ*gld1* mutant displays significantly elevated Na^{+} influx compared to wild type (WT) and the complemented strain (*Δgld1::gld1*), with minor rescue upon exogenous glycerol addition (*Δgld1* + glycerol) (n = 6 biological replicates). **B**, Intracellular glycerol content during salt adaptation, quantified as micro mol glycerol per gram of dry hyphal weight. In WT and *Δgld1::gld1*, glycerol rapidly accumulates within 8 h of NaCl exposure and then gradually declines. In contrast, *Δgld1* shows no detectable induction of intracellular glycerol, confirming its functional loss in osmolyte biosynthesis (mean ± SD, n = 3 biological replicates). **C**, Growth assays on PDA supplemented with 0.8 M NaCl and increasing concentrations of exogenous

glycerol (0.5–4%). Exogenous glycerol partially rescued growth of *Δgld1* but not to WT levels. **D**, Relative expression of *gld1* in WT in response to NaCl stress supplemented with glycerol (0.5–4%), showing marked downregulation compared to NaCl-only control (mean ± SD, n = 3; ***$P < 0.001$). Statistically significant differences between glycerol added samples and the control are indicated by stars, Student's t test, *, $P < 0.05$; **, $P < 0.01$; ***, $P < 0.001$.

Supplementary Figure 5 | Fold-change expression of osmotic stress-related genes under ionic, osmotic, and oxidative stress in *T. asperelloides*

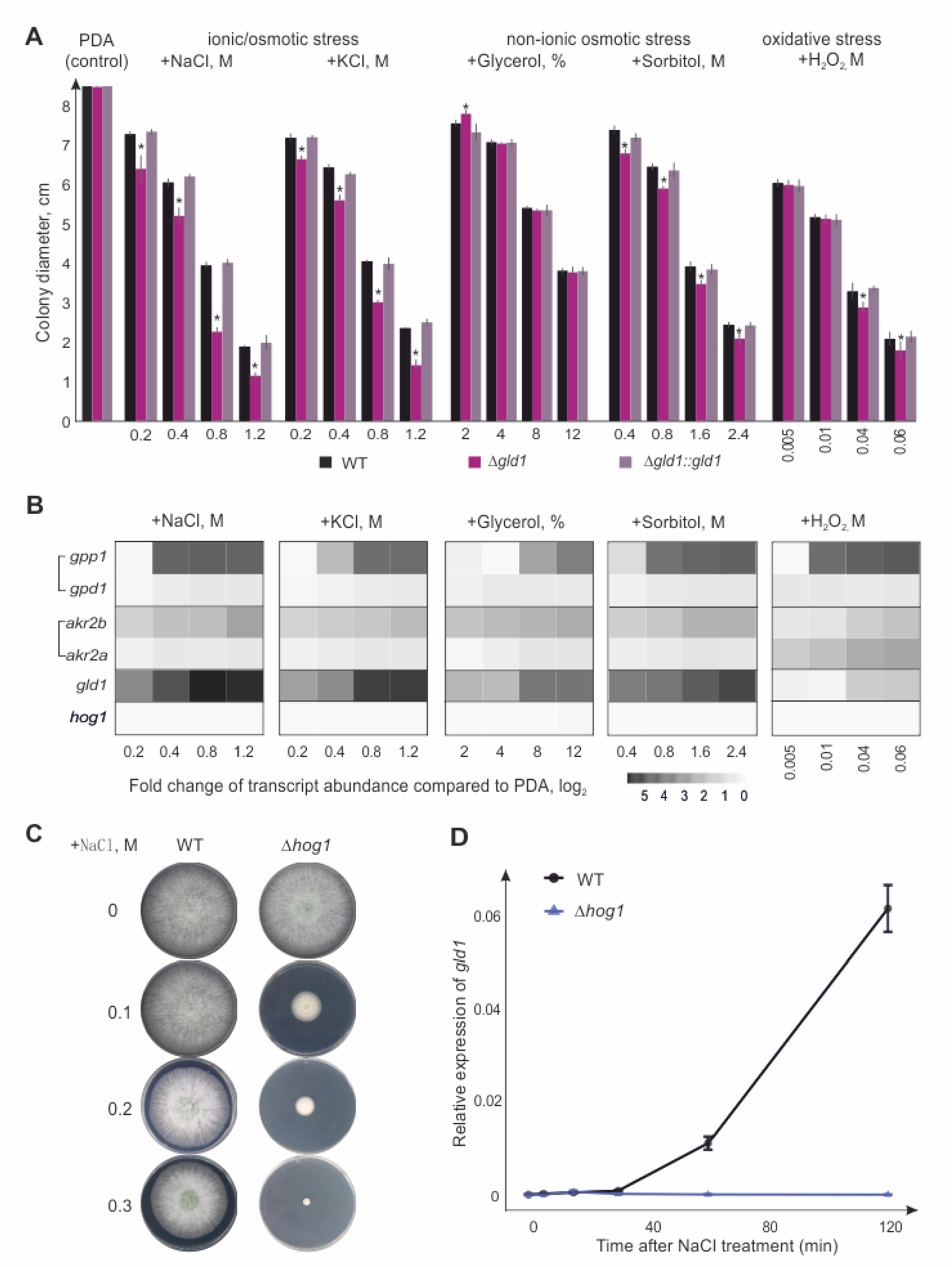

A, Colony diameter of the wild-type strain of *T. asperelloides* (black), *Δgld1* (magenta), and *Δgld1::gld1* (purple) grown on PDA or PDA supplemented with increasing concentrations of NaCl, KCl, glycerol, sorbitol, or H_2O_2. *Δgld1* exhibited salt-specific growth inhibition, which was restored in the

complemented strain. Supplementation with exogenous glycerol partially rescued the growth of *Δgld1*, particularly at lower concentrations. Statistically significant differences between *Δgld1* and WT are indicated by a star (Student's t test, $P < 0.05$). **B**, Heatmap showing $\log_2$-transformed fold changes in transcript levels of six genes (*hog1/tmk3*, *gld1*, *akr2a*, *akr2b*, *gpd1*, *gpp1*) in the wild-type strain of *T. asperelloides* under increasing concentrations of five stressors: NaCl and KCl (ionic/osmotic stress), glycerol and sorbitol (non-ionic osmotic stress), and H_2O_2 (oxidative stress). Values represent fold induction relative to PDA (unstressed control). Expression was measured by RT-qPCR, normalized to the reference gene (*tef1*), and averaged over biological replicates. *gld1* shows strong and specific induction by ionic/osmotic stress; *akr2a* is oxidative-specific, *akr2b* moderately responsive to all stressors, *gpp1* broadly inducible, *gpd1* largely constitutive, and *hog1* shows limited induction consistent with post-translational regulation. **C**, Growth of WT and *Δhog1* strains on PDA supplemented with increasing NaCl concentrations. Δ*hog1* was hypersensitive, failing to grow above 0.2 M NaCl, whereas WT tolerated up to 0.3 M. **D**, Relative *gld1* transcript abundance in WT and *Δhog1* strains following NaCl exposure, showing strong induction of *gld1* in WT but abolished expression in *Δhog1*.

**Supplementary Figure 6 | **Time-course expression dynamics of *gld1* in *Trichoderma* lineages under osmotic stress

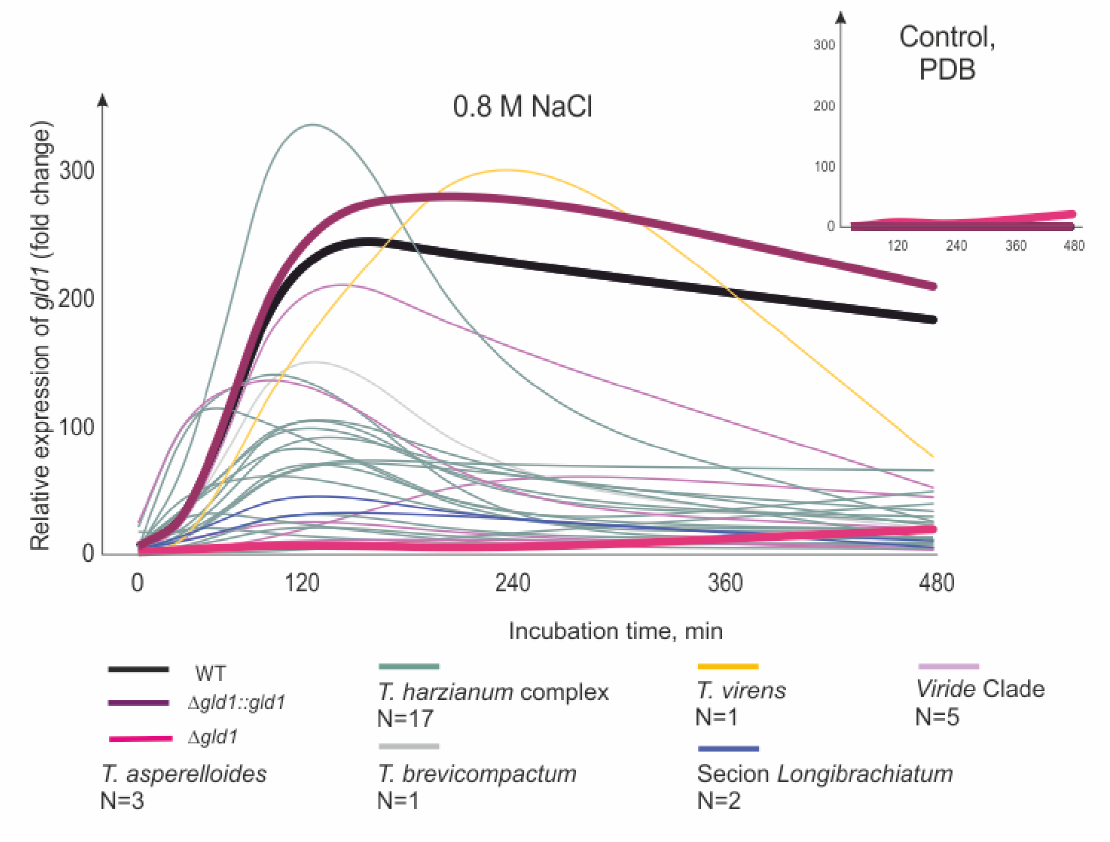

Relative *gld1* transcript abundance following exposure to PDB supplemented with 0.8 M NaCl. Each line represents a strain included in the synthetic microcosm experiment (sum = 29, **Fig. 4**). The inset shows *gld1* expression in PDB without NaCl (control), where transcript levels remained negligible. *gld1* was salt-inducible in all tested strains but varied in magnitude and duration, with *T. asperelloides* maintaining elevated expression throughout the 8 h time course. WT = wild type of halotolerant *T. asperelloides*; Δ*gld1* and Δ*gld1*::*gld1* denote mutants used in functional assays. Data are mean values (n=3).

Supplementary Figure 7 | Relative glycerol accumulation in *Trichoderma* lineages under osmotic stress

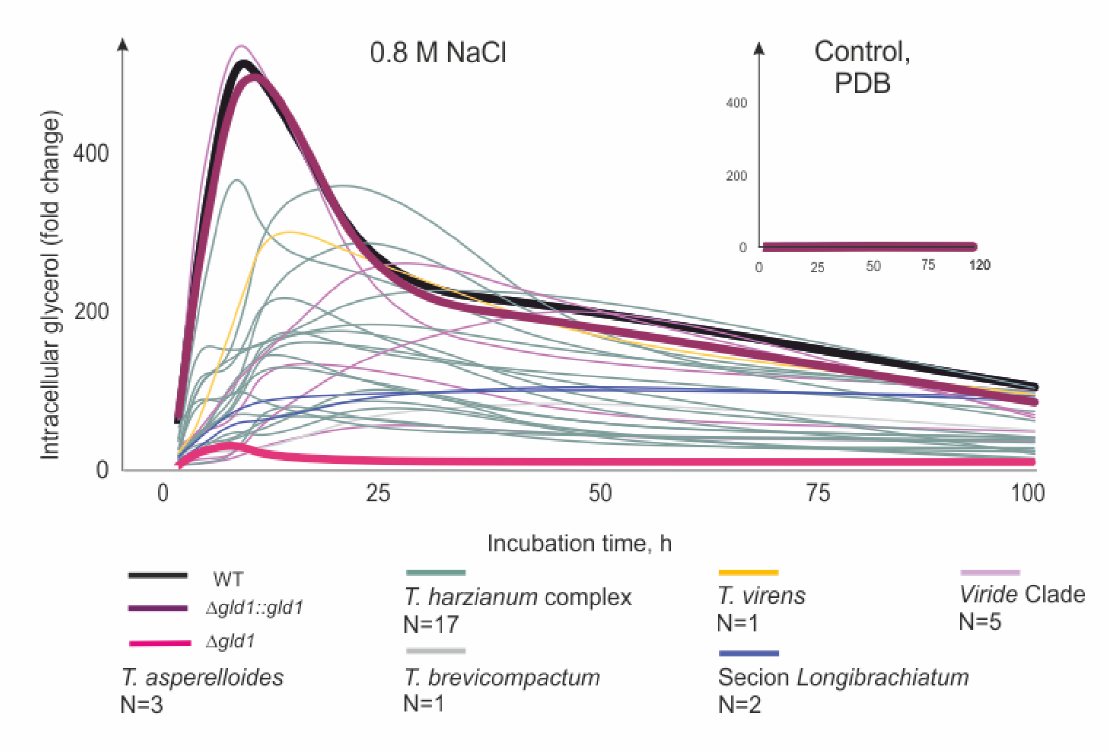

Intrahyphal glycerol levels over 96 h in 29 strains used for the synthetic microcosm experiment (**Fig. 4**) (*Harzianum* clade, N = 17; *T. virens*, N = 1; *T. brevicompactum*, N = 1; *T. asperelloides*, N = 3 including two mutants; *Viride* clade, N = 5; and section *Longibrachiatum*, N = 2), cultivated on PDA supplemented with 0.8 M NaCl. Wild-type *T. asperelloides* (black), Δ*gld1* (magenta), and Δ*gld1*::*gld1* (purple) are highlighted for comparison with other taxa. Although most species showed transient glycerol accumulation, *T. asperelloides* sustained elevated levels and coupled this response with growth under salt stress, consistent with its halotolerant phenotype. The inset shows negligible glycerol accumulation in the same strains grown on PDA without NaCl (control). Data represent mean values (n=3).

Supplementary Figure 8 | Validation of the *rpb2* amplicon for species-level profiling of the synthetic *Trichoderma* community

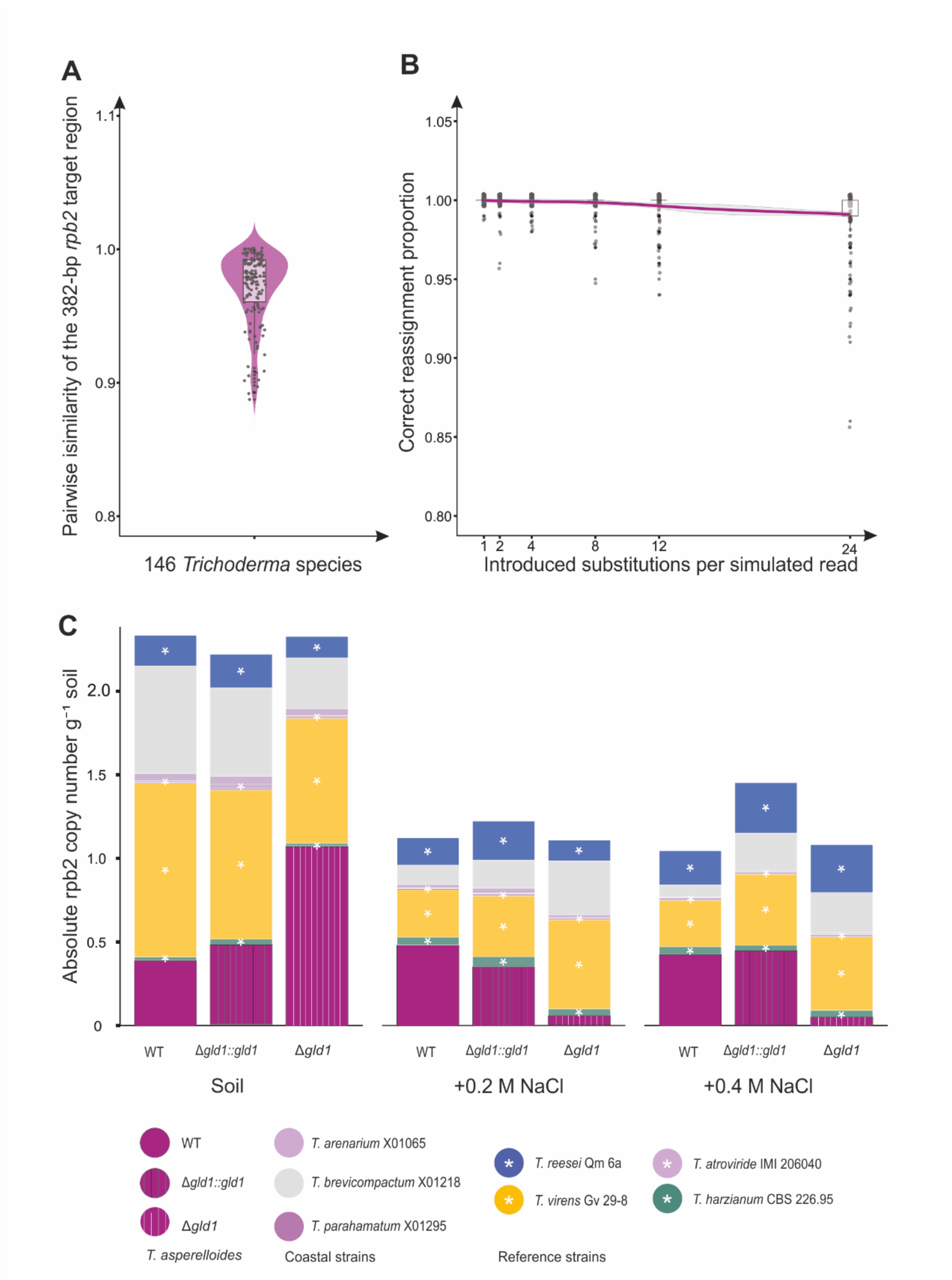

A, Pairwise sequence similarity distribution for the 382-bp *rpb2* target region across 146 *Trichoderma* species, including all species represented in the synthetic community. **B**, *In silico* reassignment robustness of the *rpb2* target

region. Simulated reads were generated by introducing 1–24 substitutions into randomly selected reference sequences at polymorphic sites, with 1000 simulations per mutation level, followed by reassignment against the same local reference database and thresholds used for the amplicon data. Reads with up to eight substitutions were reassigned correctly in >99.8% of the total cases. **C**, Experimental comparison of Illumina *rpb2* amplicon profiles and species/strain-specific TaqMan probe qPCR profiles for eight representative community members. The corresponding alignments, pairwise identity matrix, simulation outputs, and abundance data are provided in **Supplementary Data 5**.
