# Supplementary material for: Regulatory adaptation of an accessory gene controls fungal halotolerance and niche expansion
Source: ISME J. 2026 Jun 26;20(1):wrag162. doi: 10.1093/ismejo/wrag162 (PMC13452454; doi:10.1093/ismejo/wrag162)
Supplement: Supplementary file 1 [file supplementary_material_wrag162.zip › Ding et al SI Data 5.zip!/in silico simulation.pdf]

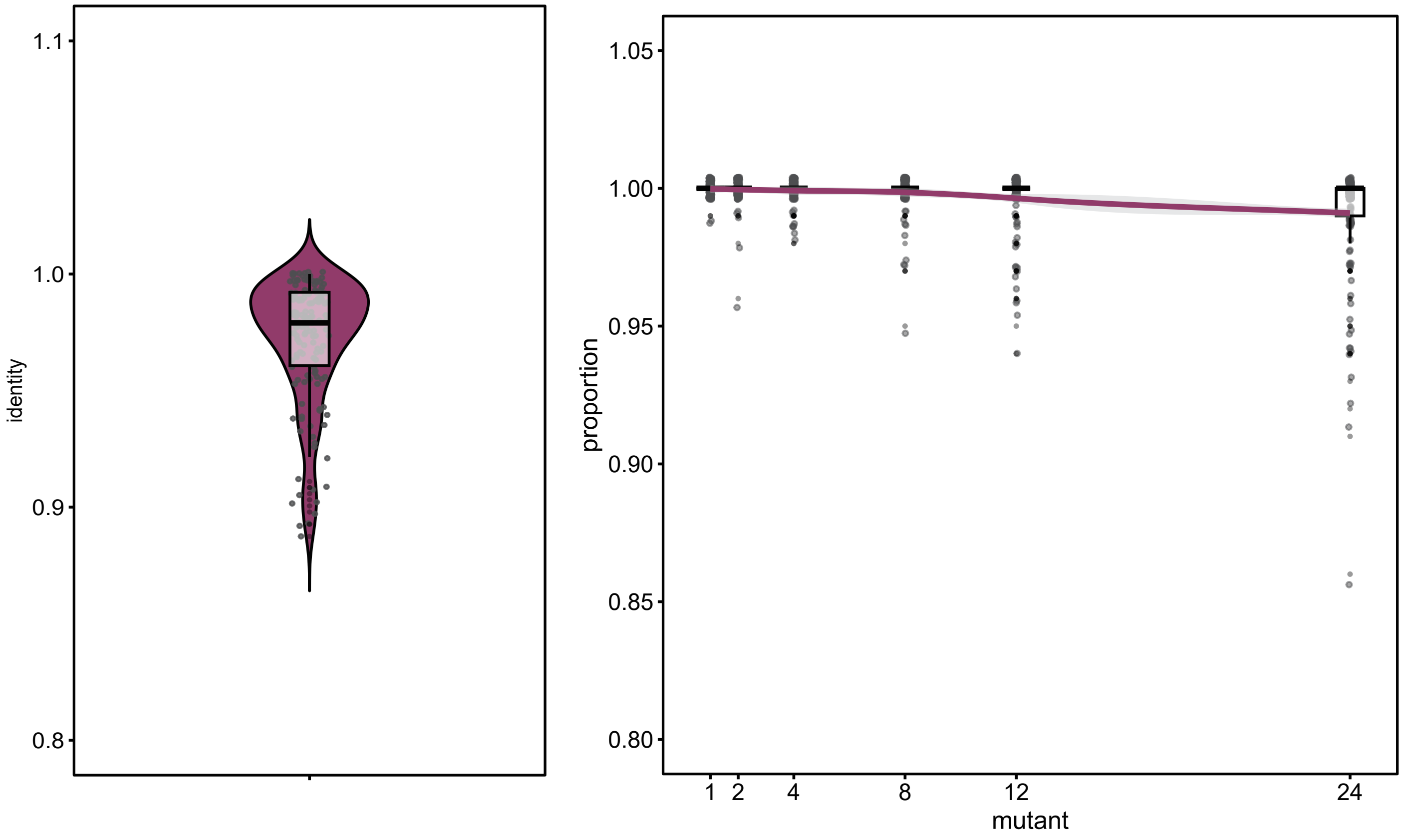

1.1
1.0
0.9
0.8
identity
1.05
1.00
0.95
0.90
0.85
0.80
proportion
1
2
4
8
12
24
mutant
